# PSO-DT based BagDT: a robust lightweight ensemble framework for efficient feature selection and DDoS attack detection in IoT environment

**DOI:** 10.1038/s41598-025-20175-7

**Published:** 2025-10-16

**Authors:** J. Jasmine Shirley, M. Priya

**Affiliations:** School of Computer Science Engineering and Information Systems, Vellore Institute of Technology, Vellore, 632014 India

**Keywords:** DDoS attack, Ensemble learning, Feature selection, Internet of things, Particle swarm optimization, Risk factors, Engineering

## Abstract

The recent decade has seen enormous growth in the Internet of Things field. This development has significantly expanded the space for cyber-threats, among which the Distributed Denial of Service attacks have become one of the most important and common threats. These attacks might severely disrupt critical services if not detected and handled on time. To provide a reliable and secure IoT environment, accurate and effective mechanisms for detecting DDoS attacks in real-time are the most required. While state-of-the-art deep learning models like CNNs and LSTMs offer high accuracy, their computational overhead often makes them unsuitable for resource-constrained IoT environments. To address this gap, we have proposed a robust hybrid framework, the PSO-DT-based BagDT ensemble model. This model utilizes Particle Swarm Optimization in combination with Decision Tree for effectively finding the best feature subset. This lowers the dimension by reducing complexity. The proposed PSO-DT feature selection algorithm is evaluated across variants of ensemble learners, namely Random Subspace KNN, AdaBoost, RUSBoost, and Bagged Decision Trees. The PSO-DT helps in reducing the computational cost and the model size. Our PSO-DT based Bagged DT model demonstrates superior performance, achieving an accuracy of 99.96 % along with a macro-average precision, recall, and F1-score of 0.99. Among all the variants, BagDT performed better with an increase in accuracy by 4.13% and a reduction in training time by 95.49%. The overall throughput is increased by 63.52% thereby confirming the efficiency of the proposed PSO-DT-based BagDT Ensemble model for providing a real-time, scalable solution that is appropriate for implementation in contemporary smart environments.

## Introduction

Advancements in technologies like Internet of Things (IoT), Artificial Intelligence (AI), Machine Learning (ML), and Deep Learning have revolutionized how devices interact with each other in the real-world environment. This has widely encouraged industrialists to develop and deploy multiple applications across smart cities, healthcare, agriculture, finance, and home automation. Although there is a positive transformation in the environment, IoT devices are resource-constrained as well as heterogeneous in nature and also lack robust security features. This nature makes them the primary target for cyber threats, especially Distributed Denial of Service (DDoS) attacks. DDoS attacks are those in which the network and the servers are flooded with illegitimate traffic with the help of multiple compromised devices or bots. In certain cases, the IoT devices are hijacked and made to act as botnets like Mirai, Mozi or Torii. These can then be used in launching large-scale DDoS attacks, which can paralyze the infrastructure, disrupt services and hence cause economic damage.

In 2024, a survey was undertaken by Zayo on the insights of DDoS attacks. The report revealed that there was a staggering increase of about 82% in DDoS attacks from 2023 to 2024. The incidents increased from 90,000 to 165,000 attacks^1^. The key insights are as follows.

- The healthcare industry experienced a 223% rise in DDoS attacks as a result of the huge amount of interconnected medical devices.
- The finance and telecom sectors were found to be the most targeted ones due to their nature of high availability.
- The average cost estimated as of now is 2,34,000 dollars per DDoS attack if it lasts for a time frame of 39 minutes, which amounts to 6,000 dollars per minute.

The various DDoS attacks that occurred in 2024 alone are as follows.

- In East Asia, a smart city transportation system was brought to a halt due to the SYN flood attack, which is a type of DDoS attack that targeted the traffic lights and surveillance feeds.
- The European manufacturing plants were disrupted by the UDP flood attacks, which exploited the insecure Industrial IoT gateways.
- In North America, one of the public utility grids was temporarily disabled due to a DDoS botnet that exploited the default user credentials that were used in smart meters.

These reports and surveys not only portray the scale at which DDoS attacks occur but also portray the diversity of the attack, ranging from TCP SYN floods, UDP floods, and ICMP fragmentation to application-layer attacks such as HTTP floods and Slowloris. These challenges make the traditional detection systems fail in real-time. Moreover, IoT also poses certain challenges that are unique, such as Heterogeneous Protocols, limited resources, high class imbalance and dynamicity of attacks. To handle these challenges, a dynamic attack detection mechanism that can adapt to the requirements needs to be deviced. This motivates researchers across the world to focus on developing ML-based DDoS attack detection schemes and frameworks. Having these in mind, this work focuses on developing an integrated framework based on the ensembling of various machine learning classifiers. The base weak learners are combined and boosted to build a strong prediction model that can adapt according to the situation.

### Novelty and contributions

While many studies have applied machine learning to DDoS detection, a significant portion focuses on maximizing accuracy with complex models such as deep neural networks that are ill-suited for the computational and memory constraints of real-world IoT devices. Our research addresses this critical gap by focusing on a lightweight, deployment-ready framework without sacrificing performance. The primary innovations and contributions of this work are as follows

- **A Novel Hybrid Feature Selection Algorithm:** We introduce a unique feature selection algorithm, PSO-DT, which combines Particle Swarm Optimization with a Decision Tree evaluator. Unlike conventional uses of PSO for hyperparameter tuning, our approach employs it as a wrapper-based feature selector to discover the optimal feature subset. This method significantly reduces data dimensionality, which in turn lowers model complexity and computational cost.
- **Emphasis on Deployment Readiness and Scalability:** A key novelty of this paper is the rigorous evaluation of deployment feasibility. We move beyond traditional classification metrics to analyze and optimize for model size (MB), prediction speed (observations/sec), and training time. This focus ensures our proposed model is not just a theoretical success but a practical solution for real-time detection on edge devices.
- **An Integrated and Holistic Framework:** This work presents a complete, end-to-end framework that cohesively integrates solutions for common challenges in IoT security datasets. It combines automated handling of class imbalance using SMOTE, the novel PSO-DT feature selection, and an efficient Bagged Decision Tree (BagDT) ensemble classifier, resulting in a robust and reproducible system.

The rest of the paper is structured as follows. Section “Related works” reviews the works related to DDoS attack detection carried out by researchers in recent years across the world. Section “Background fundamentals” provides a brief introduction to the fundamentals and background needed for a better understanding. Section “Proposed methodology” describes in detail the proposed feature selection algorithm PSO-DT, the integrated framework and the ensemble prediction model PSO-DT based on BagDT. Section “Results and discussions” presents a detailed description of the results obtained for each variant and the comparative analysis of the complete experiment carried out. The paper is finally concluded with challenges, scope and future enhancements.

## Related works

The recent decade has seen multiple researchers working on developing novel frameworks for detecting cyber attacks using ML and DL methods. This section discusses the contributions of a few researchers who have performed similar experiments on the data set CICIoT2023 and other similar types. The authors in^2^ aimed to develop a prediction model for detecting cyber attacks using hybrid ML models. Two ensemble techniques were experimented on the dataset, namely voting and stacking, using Logistic Regression (LR), Gaussian Naive Bayes (GNB), and Random Forest (RF) algorithms are used as base learners. Their model utilised pre-processing steps, and hyperparameter optmization was done for improving the performance of the hybrid ML model. The voting-based ensemble learner produced 99% accuracy for binary class classification and 98% for multiclass. Their study did not focus on deployment feasibility, computational complexity and scalability. In^3^, the researchers had proposed an ensemble model using multiple base classifiers, namely LR, Naive Bayes (NB), Support Vector Machine (SVM), K-Nearest Neighbour (KNN) and Multi-Level Perceptron (MLP). They experimented with their proposed ensemble learner on two data sets, namely CICIoT2023 and Edge-IIoTset2023. Experiments were also conducted using variants of ensembling techniques, voting, bagging, boosting and stacking. They interpreted the performance of the ensemble learner using the metrics precision, recall, F1 score and accuracy. They concluded that the boosting-based ensemble technique provided better accuracy of 93% when compared to other variants. These researchers also focused only on measuring the performance of the proposed ensemble model on both data sets where whereas they did not focus on computational complexity or deployment feasibility.

Further expanding on deep learning approaches, Sumathi et al.^4^ proposed a hybrid model using Long Short-Term Memory (LSTM) and a hybrid Harris Hawks-PSO (HHO-PSO) algorithm for feature selection, demonstrating the power of combining bio-inspired optimization with recurrent neural networks. In a comparative study, the efficacy of several machine learning algorithms for detecting specific attack vectors like the TCP SYN flood was analyzed, highlighting the trade-offs between different models^5^. Other works have also explored deep learning methods^6^ and hybrid frameworks to address the evolving threat landscape in IoT environments. A common thread in much of this research is the focus on sophisticated deep learning architectures, which, while powerful, often do not prioritize the lightweight and deployment-ready characteristics essential for resource-constrained IoT devices^7^ .

**Table 1:** Summary of related works.

Ref.	Contribution	Dataset for validation	Performance analysis	Limitations
^2^	Used Hybrid ML Ensemble (Voting, Stacking) of LR, GNB, RF	CICIoT2023	Accuracy: 99% (binary), 98% (multi-class)	No focus on deployment feasibility, computational cost, or scalability
^3^	Developed an Ensemble model with LR, NB, SVM, KNN, MLP using Voting, Bagging, Boosting, Stacking variants	CICIoT2023, Edge-IIoTset2023	Boosting achieved 93% accuracy	Lacked analysis on deployment feasibility and efficiency
^4^	Compared Individual ML/DL vs. Ensemble (DNN, RF, DT)	CICIoT2023, WSN-DS	Ensemble outperformed standalone models	Focus limited to accuracy, no deployment considerations
^5^	Developed a Hybrid Stacking Ensemble and Cuckoo Search Optimization	CICIoT2023, KDDCup99, CSE-CIC-IDS2018	Accuracy: 99.87% (binary), 98.86% (multi-class)	Deployment and complexity not addressed
^6^	Used Voting Ensemble with RF as Base Model	CICIoT2023	F1-Score: 99.75%, FAR: 0.038%	No scalability or real-time feasibility assessment
^7^	Combined Recursive Clustering, Entropy Sampling and ML classifiers	CICIoT2023	High precision, recall, and F1-score	Focused mainly on sampling and feature selection
^8^	Developed a Hybrid deepCLG (CNN, LSTM, GRU, Capsule Net)	CICIoT2023, UNSW_NB15	Accuracy: 99.82%	No attention to resource constraints or deployment aspects

In^8^, the authors compared the performance of individual ML and DL models with the ensemble learner for detecting DDoS and DoS attacks. The three models used in their study are deep neural network (DNN), RF, and decision tree (DT). The performance metrics utilised were precision, recall, F1-score and accuracy. They grouped the total number of 33 attacks in the CICIoT2023 data into 7 major classes for performing the study. The researchers also performed the study on another dataset set namely WSN-DS, which contained four types of DoS attacks. Their experiments concluded that the ensemble approach achieved better accuracy when compared stand stand-alone ML and DL approaches. The authors in^9^ proposed an integrated framework for enhancing the security in an IoT environment using a stacking ensemble approach. To reduce the dimension, they used the Cuckoo search optimization algorithm. They tested their proposed ensemble model on various data sets, which include KDD Cup 99, CSE-CIC-IDS2018, and CICIoT2023. The maximum accuracy achieved on binary classification was 99.87% on the KDD Cup 99 data set. In the case of the multiclass approach, the accuracy achieved was 98.86%. In^10^, the researchers experimented with an ensemble approach based on a voting mechanism. The base learner utilized in this study was RF. The model was experimented on the CICIoT2023 data set. Their model achieved an F1 score of 99.75%. They showed a false alarm rate of 0.038%. In^11^, the authors proposed a recursive clustering method based on K-means for condensing the data and used an entropy-based sampling method for preparing an apt subset of the large data set. They utilized the Monte Carlo entropy technique for selecting the relevant features. The classification was performed using various classifiers, namely RF, LR, KNN, J48, NB and LinearSVC. They utilized precision, recall and F1 measure as evaluation metrics. Their major focus was on developing an effective sampling and feature selection technique for large data sets.

The researchers in^12^ focused on Industrial IoT networks and developed a framework for detecting the traffic irregularities. They proposed a hybrid model named deepCLG. The model is a hybrid version that utilized CNN, long short-term memory (LSTM), and the gated recurrent unit (GRU) and integrates these with a capsule network. The authors analyzed the efficiency of the proposed model on two data sets, CICIoT2023 and UNSW_NB15. The model achieved an accuracy of 99.82%. The authors in^13^ expanded on this research by developing an adaptive intrusion detection system that integrates autoencoders with a feedforward neural network (FNN). This innovative approach demonstrated impressive classification performance against emerging IoT threat vectors. Their Autoencoder-FNN fusion model emphasized effective feature extraction and dimensionality reduction while ensuring high detection accuracy. A summary of the contributions and limitations of the works carried out by the researchers in the same domain is illustrated in the Table 1. The last decade has seen an increasing trend in leveraging ensemble machine learning and deep learning techniques for cyber attack detection, particularly in IoT environments. Most studies have shown that ensemble approaches, especially stacking, voting, and boosting, outperform individual ML or DL models in terms of detection accuracy, precision, recall, and F1-score. Specifically, accuracy values have consistently exceeded 98% in many cases, with some hybrid models even achieving performance close to 99.8%. However, though performance metrics are well explored across these experiments, a common limitation is the lack of attention to deployment feasibility, computational efficiency, and scalability. These aspects are crucial for real-time IoT security systems, especially when operating under constrained environments such as edge or fog computing. The Table 2 illustrates how our proposed framework is unique from other related works discussed in this section.

**Table 2:** Comparative analysis of contributions of proposed work vs related works.

Aspect	Proposed work	Cited studies
Feature Selection	Optimized PSO-DT algorithm for reducing model complexity	Mostly traditional feature selection or none. Some used entropy-based or Cuckoo search
Model Type	Hybrid predictive model (PSO-DT + BagDT) for DDoS attack classification in IoT environment	Majority used stacking, voting, boosting with common ML/DL models (e.g., RF, SVM, DNN)
Class Imbalance Handling	Uses integrated resampling, encoding and ensemble learning for addressing class imbalance	Limited or no explicit handling of class imbalance
Comparative Analysis	Provides detailed evaluation of multiple ensemble variants like AdaBoost, RUSBoost, BagDT, Random Subspace KNN in terms of accuracy, training time, prediction time and deployment feasibility	Focused mostly on accuracy, with limited or no comparison across ensemble variants
Deployment Readiness	Assesses model size, prediction speed (obs/sec), and scalability for real-time use	Rarely addressed, most studies lacked focus on deployment feasibility or real-world constraints
IoT Specificity	Explicitly designed for IoT environments with network traffic data and real-time constraints	Used IoT datasets like CICIoT2023, but lacked emphasis on real-time or resource-aware deployment
Performance Metrics	Evaluates both predictive accuracy and operational metrics like speed and scalability	Mainly used accuracy, precision, recall, F1-score, rarely used system level metrics

## Background fundamentals

This section provides a brief description of the various fundamental concepts and background required for understanding the proposed framework. The concepts discussed are Bio-inspired algorithms, Ensemble Learning and Particle Swarm Optimization techniques.

### Bio-inspired algorithms

The Bio-inspired algorithms are the methods inspired by the natural behaviour, structure and evolution of biological systems in nature. These algorithms are best suited for solving complex and dynamic problems that cannot be solved by mathematical approaches. Usually, researchers use these algorithms for non-linear high-dimensional data^14^. The major characteristics of these algorithms are as follows.

- Since these are nature-inspired, they can adapt themselves to the dynamic environment automatically.
- They generally work in a decentralized manner.
- Since randomness is the key, local minima issues are handled efficiently.
- They are all self-organised, and the solutions are derived from their local behaviours and interactions among the population.

The bio-inspired algorithms can be classified as Evolutionary Algorithms (EAs), Swarm Intelligence Algorithms, Artificial Immune Systems (AIS) and Neural and Cellular-Inspired Models. The EAs are based on the principle of Darwin, that is, survival of the fittest, which is simulated by operations like selection, mutation, crossover and reproduction^15^. Few well-known examples are Genetic algorithms^16^, Genetic programming^17^ and Differential evolution^18^. The major applications are in feature selection, training of neural networks and hyperparameter tuning. Swarm Intelligence algorithms are designed by mimicking the collective behaviour of animals like honey bees, ants, birds, lions, whales, fireflies, etc., for achieving an objective. Few popular algorithms are Particle Swarm Optimization (PSO)^19^, Ant Colony Optimization (ACO)^20^, Artificial Bee Colony (ABC)^21^ and Firefly Algorithm^22^. The major applications are in sensor networks for routing problems, detection of cyber attacks and optimized resource allocation. AIS are those techniques that are based on the principle of the human immune system. The inspiration is in the manner the human immune system detects pathogens and neutralizes them. Neural and Cellular-Inspired Models are based on the principle of intelligence at the cellular and brain level. Cellular automata^23^ and Spiking Neural Networks^24^ are well-known examples of these types. The major applications are in image and signal processing, pattern matching and temporal anomaly detection. A summary of the popular bio-inspired algorithms is illustrated in the Table 3. These algorithms can be utilized for a wide range of optimization problems and are domain-independent. They are capable of handling noisy, incomplete as well and dynamic data. Though they have various advantages, they suffer from a few drawbacks like the requirement of high computational resources, complex parameter tuning, slower convergence rate and the global optimum is not guaranteed.

**Table 3:** Summary of popular bio-inspired algorithms.

Type of Algorithm	Inspiration	Strengths	Applications
Genetic Algorithm	Natural Evolution	* Global Search * Flexible	* Feature Selection * Optimization problems
Particle Swarm Optimization	Bird/Fish Swarming	* Fast convergence * Simple	* Weighted Sum
Ant Colony Optimization	Ant Foraging	* Very good for paths and combinatorial	* Routing * Scheduling
Artificial Bee Colony	Bee Foraging	* Balanced exploration and exploitation	* Resource optimization
Artificial Immune System	Immune Response	* Anomaly detection * Memory usage	* Network security * Secure IoT environment
Firefly / Bat Algorithm	Light/echolocation	* Continuous optimization	* Signal classification * Training ML models
Cellular Automata, Spiking Neural Networks	Cells/Neurons	* Local rules * Spatiotemporal modeling	* Complex systems modeling

### Ensemble learning

Ensemble Learning (EL) is an advanced ML paradigm, where the output of various weak learners or base models is combined to improve the performance of the final prediction model. This final prediction model is usually a strong learner that combines all the best measures of individual models and is also robust. The reason behind this improved performance is that the variance and bias are reduced, thereby improving the prediction accuracy due to diversity^25^. EL techniques can be of four different types, namely bagging, boosting, stacking and voting. Bagging is a technique in which the data set is divided into multiple subsets, and the training is performed individually on each subset. This mechanism helps in reducing the variance and hence avoids overfitting. The popular base learner used here is the RF or DT. In the case of boosting, the models are utilized to train the dataset sequentially, one after the other. Each model tries to correct the mistakes made by the previous model. This helps in reducing bias, and hence performance of the final model is improved. Usually, algorithms like AdaBoost, Gradient Boosting Machines (GBM), XGBoost, LightGBM and CatBoost are used. In the case of Stacking, the base models are usually heterogeneous. The output of these models is combined using a meta-model, which is usually a weak learner. The outputs of the base learners are used as features for the meta-model. Popularly used meta-learners are LR or a multi-layer perceptron. Voting is a very simple mechanism for combining the outputs of multiple classifiers. There are two types of voting, namely hard and soft voting. In hard voting, the majority output is considered to be the final prediction. In case of soft voting, the final prediction is the average result. A summary of the various EL techniques is illustrated in the Table 4. The major challenge in EL is that they are usually computationally expensive, as multiple models need to be trained. Also, hyperparameter tuning is a challenge.

**Table 4:** Summary of ensemble techniques.

Method	Focus	Base learners type	Aggregation scheme
Bagging	Variance	Homogeneous	Averaging/voting
Boosting	Bias	Homogeneous	Weighted sum
Stacking	Generalization	Heterogeneous	Meta-learner
Voting	Simplicity	Anything	Majority/average

### Particle swarm optimization

PSO is an algorithm inspired by the swarming behaviour observed in birds, fish and other social animals^26^. Birds usually flock together to find the presence of food efficiently. Similar to that, in PSO, the particles are initialized to collaborate for finding the solution in an optimized manner. The algorithm was developed by Kennedy and Eberhart. The algorithm works on various terminologies, which are listed as follows.

- **Swarm:** This term represents the group of particles.
- **Particle:** This represents the possible solution for the given problem.
- **Position:** This represents the current location of the particle *n* and can be represented as $y_n$.
- **Velocity:** This is the speed at which the particle moves in a particular direction and can be represented as $v_n$.
- **Personal Best:** When the particle moves in search of a solution, the best solution or position of the particle *n* is specified as personal best and can be represented as $p\alpha$.
- **Global Best:** Out of all the solutions found by each particle in the swarm, the best solution is considered as the global best and is represented by $g\alpha$.

The entire algorithm is executed for multiple iterations, and the possible solutions are obtained. At every iteration, the position and velocity of every particle are updated using the following equations. The velocity of the particle can be updated using the Eq. (1). In the Eq. (1), $\omega$ represents the balance between exploitation and exploration that is termed as inertia weight, $a_1$ and $a_2$ are the acceleration constants, and $ran_1$ and $ran_2$ are the random numbers generated between the interval 0 and 1.

1 $$\begin{aligned} v_n (x + 1) = \omega .v_n(x) + a_1.ran_1.(p\alpha _n - y_n(x)) + a_2.ran_2.(g\alpha - y_n(x)) \end{aligned}$$

The position of the particle can be updated or modified using the Eq. (2).

2 $$\begin{aligned} y_n(x+1) = y_n(x) + v_n(x+1) \end{aligned}$$

Initially, a swarm of particles are initialized with some random velocity and position. Then, for each particle in the swarm, the fitness value is estimated using a fitness function. Based on this, the current position of the particle is updated, and if it is better than the previous position, the $p\alpha$ is updated with the new value. Out of all the $p\alpha$ values, the best is considered to be the $g\alpha$ value. The velocity and position of the particles are updated using Eqs. (1) and (2). These steps are repeated till a stopping criterion is reached. The stopping criteria can be a maximum value for iterations or a best fitness value, which is decided by a threshold.

This research has proposed a feature selection algorithm, PSO-DT, based on the PSO algorithm, and a prediction model for detecting DDoS is designed by using an ensemble approach. All the variants are utilized for experimental purposes, and the best variant is decided. The next section discusses about the proposed methodologies.

## Proposed methodology

Due to technological advancements in ML, DL and IoT, various smart devices are being installed in real-world environments. These devices are usually installed to remotely monitor the environment and transfer the data via the internet to a centralized cloud framework where some drastic decisions are taken and actuators are initialized. These types of smart devices are usually installed as edge devices and are also heterogeneous. Though there are multiple smart applications in various domains and industries, these are not generally adopted in a wide range of real-time applications. The reason behind this hesitation is the security aspect of these devices, which are resource-constrained and hence need to transmit data over a public network. These networks and data are usually exposed to data threats and data breaches, which might lead to negative impacts. The traditional intrusion detection systems are designed based on some static patterns and cannot adapt to the current dynamic environment. Moreover, the IoT devices are usually smaller in size and lack storage and memory capacity. Hence, having a lightweight model for detecting the attack is most needed. Our proposed integrated framework, PSO-DT-based BagDT, is best suited for this scenario and is illustrated in the Algorithm 1.

**Algorithm 1:**
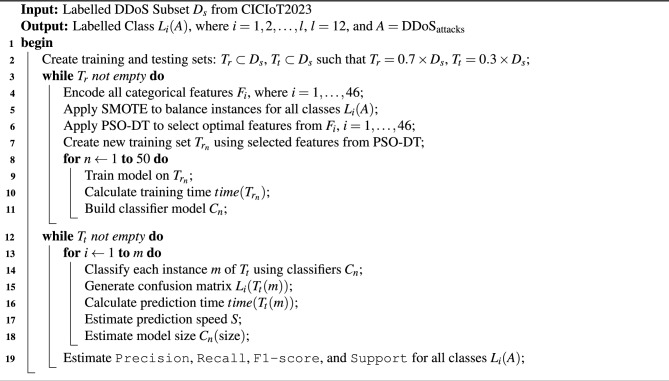
Proposed integrated PSO-DT based BagDT ensemble model

**Figure 1:**
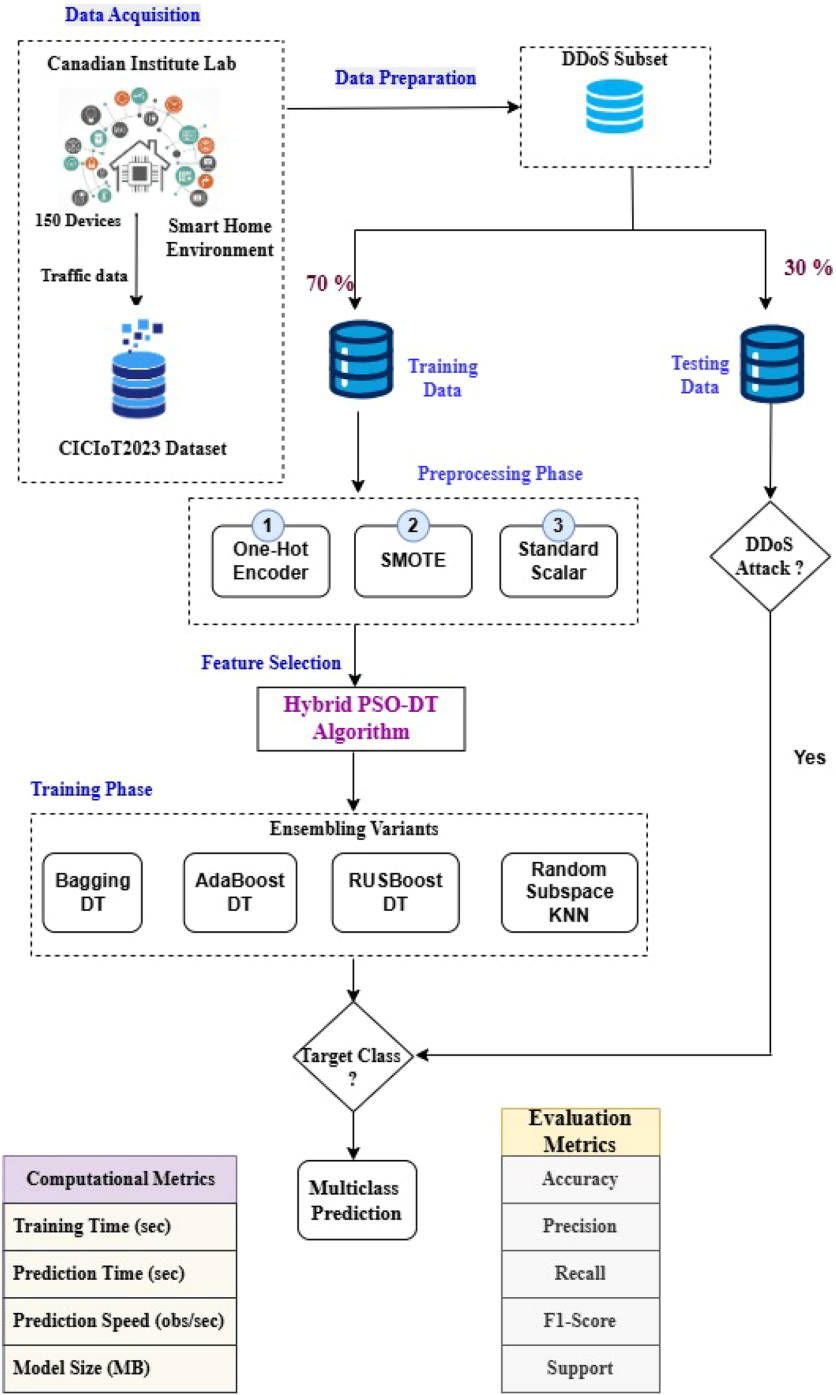
Hybrid PSO-DT based ensemble framework for DDoS attack detection in IoT environment..

The proposed integrated framework is illustrated in Fig. 1.

**Figure 2:**
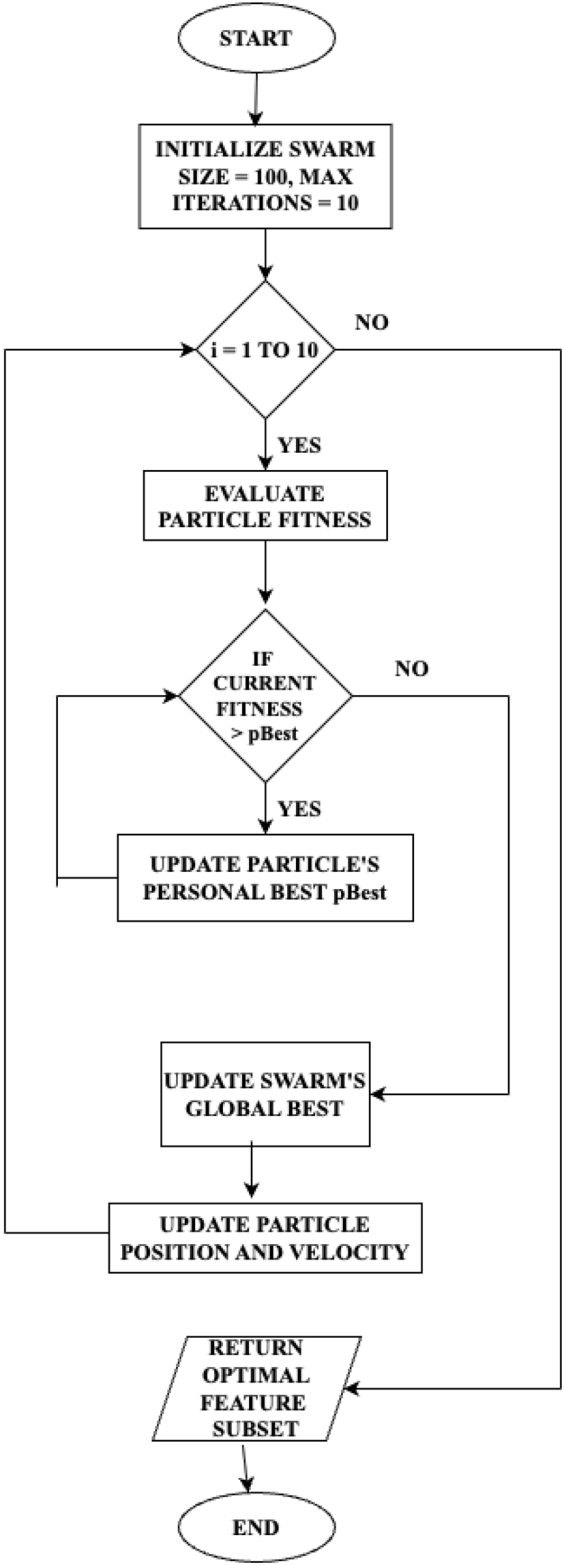
Flowchart of the PSO-DT feature selection process.

The integrated framework has a total of five phases, namely the data preparation phase, pre-processing phase, feature selection, training phase and testing phase. The data gathered from a set of 150 IoT devices in the CIC Lab is the CICIoT2023 dataset. This dataset is initially prepared in the data preparation phase, where the target classes related to DDoS attacks alone are filtered and separated to form an individual subset. This subset is then tested for NULL values. Since there were no NULL values, the subset was taken as such and split into training and validation data sets using the 70-30 rule. Then the training data set is passed over to the Pre-processing phase, which includes three components. Initially, the data is encoded using the one-hot encoding scheme to convert the categorical data available in the dataset into numerical data so that it can be further utilized for processing. During data visualization, since it was noticed that there was class imbalance, to balance the distribution of classes, the SMOTE oversampling technique is utilized. Then the balanced data set is normalized using the Standard scalar method. The preprocessed training data is then used as input for the proposed PSO-DT feature selection algorithm. The PSO-DT algorithm is illustrated in Algorithm 2. The operational logic of the PSO-based feature selection process is visually detailed in the flowchart in Fig. 2. The selected features are then used for training the four ensemble models, BagDT, AdaBoost, RUSBoost and Random Subspace KNN. The prediction models are evaluated using the evaluation metrics Precision, Recall, F1 score, Support, Training time, Testing Time, Prediction speed in terms of observations per second and model deployment size in MB. Then the models are validated using the validation data. All the models are evaluated and compared with the prediction model MRMR-PCA BagDT model, which was proposed in our previous research. The best model is decided based on accuracy, model size and computational complexity.

**Algorithm 2:**
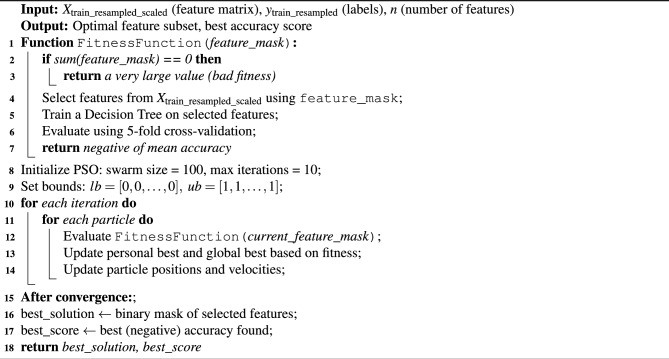
PSO-based feature selection with decision tree

## Results and discussions

This section discusses in detail the data set used to experiment, the experimental setup, the hyperparameters used, the results obtained and the discussion on the validation results.

### Dataset description

The data set utilized to validate the proposed framework is the real-time data collected at the Canadian Institute of Cyber Security (CIC). The data is generated in the CIC’s IoT lab set up specifically for producing IoT-based security data^27^. The lab was set up with a total of 150 IoT devices deployed all over the laboratory to mimic the real-world environment. Out of these, around 67 devices were utilized to create the attacks, and the remaining 38 were deployed in the network using Zigbee and Z-wave devices. These were further connected to the hubs, which were 5 in number. The various devices interconnected in the lab are smart home devices like cameras, televisions, sensors and micro-controllers. The attacks are produced using various tools and software that are installed in the environment. Various types of attacks were produced and captured, namely DDoS, DoS, Recon, Web-based, Brute-force, Spoofing and Mirai. Each type of attack had sub-sets. The details of all the classes under each category of attack are illustrated in the Table 5.

**Table 5:** Distribution of attack instances across categories and their respective target classes in the dataset.

Category of Attack	Target Classes	Number of Instances
Distributed Denial of Service	UDP Flood	5,412,287
	SlowLoris	23,426
	RSTFIN Flood	4,045,285
	HTTP Flood	28,790
	ACK Fragmentation	285,104
	ICMP Flood	7,200,504
	PSHACK Flood	4,094,755
	UDP Fragmentation	286,925
	ICMP Fragmentation	452,489
	TCP Flood	4,497,667
	SYN Flood	4,059,190
	SynonymousIP Flood	3,598,138
Denial of Service	TCP Flood	2,671,445
	HTTP Flood	71,864
	SYN Flood	2,028,834
	UDP Flood	3,318,595
Recon	Ping Sweep	2,262
	OS Scan	98,259
	Vulnerability Scan	37,382
	Port Scan	82,284
	Host Discovery	134,378
Web-based	SQL Injection	5,245
	Command Injection	5,409
	Backdoor Malware	3,218
	Uploading Attack	1,252
	XSS	3,846
	Browser Hijacking	5,859
Brute-Force	Dictionary Brute Force	13,064
Spoofing	ARP Spoofing	307,593
	DNS Spoofing	178,911
Mirai	GREIP Flood	751,682
	Greeth Flood	991,866
	UDPPlain	890,576

**Figure 3:**
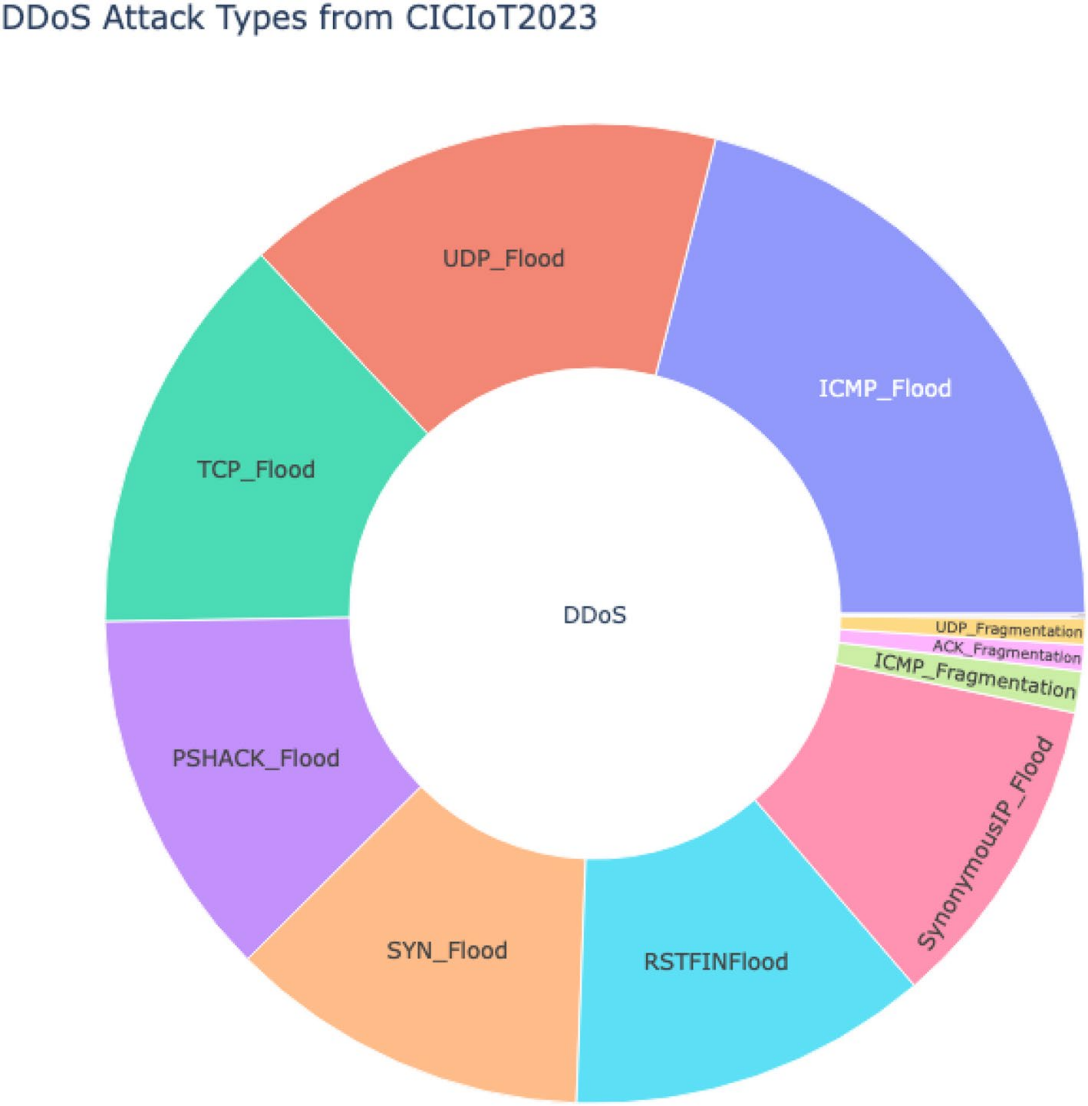
DDoS attack types for experimentation extracted from CICIoT2023.

The proposed framework concentrates on experimenting only with the DDoS category and its corresponding target classes, namely ACK_Fragmentation, HTTP_Flood, ICMP_Flood, ICMP_Fragmentation, PSHACK_Flood, RSTFINFlood, SYN_Flood, SlowLoris, SynonymousIP_Flood, TCP_Flood, UDP_Flood and UDP_Fragmentation as shown in the Fig. 3. The subset has 47 features, of which one is the target class and of categorical type. All other features are numerical and of type float.

### Experimental setup

To ensure the transparency and reproducibility of our findings, all experiments were conducted in a consistent computational environment. The hardware and software configurations used for data processing, model training, and evaluation are detailed below.

$\bullet$ All experiments were conducted within the Google Colab environment, which provided a virtual machine equipped with an Intel Xeon CPU (running at 2.20GHz or faster) and 12 GB of system RAM. For accelerated computations where applicable, a Tesla T4 or P100 GPU was utilized. To ensure efficient model training, Scikitlearn’s n jobs=-1 parameter was used to leverage all available CPU cores for parallel processing.

$\bullet$ The entire workflow was implemented in Python version 3.8+. Core functionalities were handled by several key open-source libraries, with the following versions being used.

- Pandas (v1.3.5) was used for data loading and manipulation.
- Scikit-learn (v1.0.2) was utilized for building the ensemble models namely BaggingClassifier, AdaBoostClassifier. Then also for data scaling, and performance metric calculations.
- Imbalanced-learn (v0.8.1) was for applying the SMOTE technique to correct class imbalance.
- Pyswarm (v0.6) was used for implementing the Particle Swarm Optimization algorithm.
- Matplotlib (v3.2.2) and Seaborn (v0.11.2) were utilized for generating all visualizations, including confusion matrices and performance graphs.

### Data pre-processing

**Figure 4:**
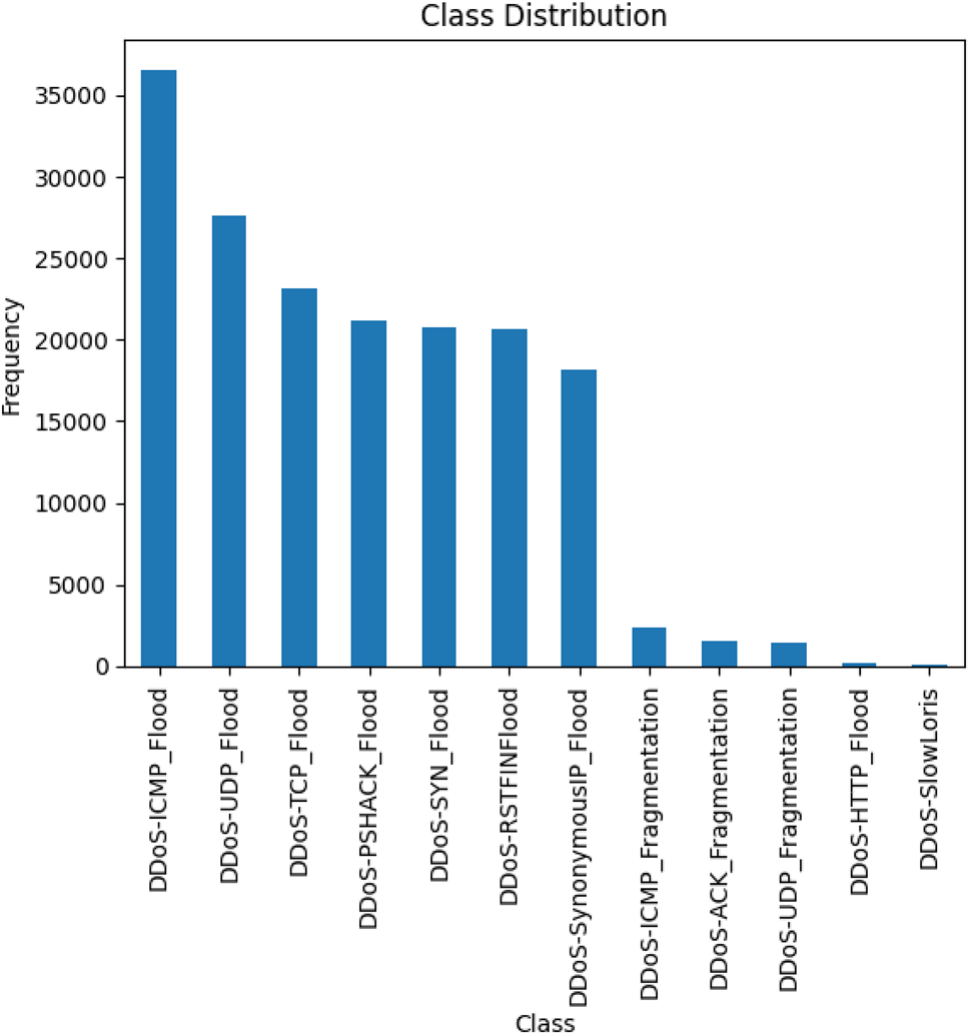
Distribution of classes across the dataset.

Data pre-processing began with an integrity check, which confirmed there were no missing values. The primary challenge identified during the initial analysis was a severe class imbalance, as illustrated in the class distribution plot in the Fig. 4.

With a calculated imbalance ratio of over 344:1, the dataset is heavily skewed towards majority classes. Training a model on such an imbalanced dataset can lead to a biased classifier that performs well on frequent attack types but fails to detect rare yet critical threats, rendering it unreliable for real-world security.

To mitigate this, we incorporated the Synthetic Minority Over-sampling Technique (SMOTE) into our framework. SMOTE was specifically chosen over simple over-sampling or under-sampling because it generates new, synthetic data points for the minority classes by interpolating between existing instances. This approach creates a more balanced and robust training set without discarding potentially valuable data or simply overfitting to duplicate samples.

Since the dataset had categorical features, we used the one-hot encoding technique to encode the categorical features. We decided to keep the numerical features as such. Hence, when numerical features were found, we used the pass-through transformer mechanism to avoid any encoding. For balancing the classes across the training dataset, we used SMOTE. A random state of 42 was initialized for performing this balancing operation. The distribution of classes before and after applying SMOTE is illustrated in Table 6. To normalise both the training and testing data, normalization was performed using the Standard Scalar mechanism.

**Table 6:** Distribution of instances for each class: before vs after SMOTE.

Target class	Before applying SMOTE	After applying SMOTE
ICMP_Flood	25,629	25,629
UDP_Flood	19,257	25,629
TCP_Flood	16,167	25,629
PSHACK_Flood	14,801	25,629
SYN_Flood	14,566	25,629
RSTFINFlood	14,538	25,629
SynonymousIP_Flood	12,753	25,629
ICMP_Fragmentation	1651	25,629
ACK_Fragmentation	1064	25,629
UDP_Fragmentation	1011	25,629
HTTP_Flood	121	25,629
SlowLoris	85	25,629

### Feature selection

The dataset had a total of 47 features, out of which one is the target class and the other 46 are features correlating to the target class. When all features were utilized for training the data, the time required for training was higher, and it would also require more computational resources. To overcome these issues, we tried to select the best features using the proposed PSO-DT framework, where only 19 features were selected. The fitness score obtained as a result of the proposed PSO-DT feature selection algorithm is illustrated in the Table 7. The algorithm tries to minimize the fitness function, which is defined. Here, since we want to maximize the accuracy, the negative accuracy is considered to be returned as a result. In the feature subset, ‘0’ represents that the feature is not selected and ‘1’ represents that the feature is selected. Hence, a binary mask is utilized. In every iteration, the selected feature subsets are evaluated using the DT classifier with 3-fold cross-validation initially and then again evaluated with 5-fold cross-validation to test the quality of the feature subset. The features which were selected as a result of this phase were Drate, syn_flag_number, rst_flag_number, ack_count, syn_count, fin_count, HTTP, SMTP, IRC, UDP, ICMP, IPv, LLC, AVG, IAT, Number, Magnitude, Covariance and Variance. These selected features were used for training the proposed ensemble models.

**Table 7:** Fitness score for features.

Features	Fitness score	Features	Fitness score	Features	Fitness score
flow_duration	0.30436109	ack_count	0.94010189	DHCP	0.21379606
Header_Length	0.13199394	syn_count	0.85842519	ARP	0.33428846
Protocol Type	0	fin_count	0.75775922	ICMP	0.99799178
Duration	0.39785963	urg_count	0.3499426	IPv	0.67377248
Rate	0.2021474	rst_count	0.35770719	LLC	0.85858186
Srate	0.49855604	HTTP	0.60686721	Tot sum	0.41871924
Drate	0.76882591	HTTPS	0.04443177	Min	0
fin_flag_number	0.15159532	DNS	0	Max	0
syn_flag_number	0.55217229	Telnet	0	Avg	1
rst_flag_number	0.59178972	SMTP	0.77489411	Std	0.11144784
psh_flag_number	0.13252155	SSH	0.16439804	Tot size	0.01078028
ack_flag_number	0.07472793	IRC	0.66806486	IAT	0.62099422
ece_flag_number	0.18252335	TCP	0.40778826	Number	0.78897386
cwr_flag_number	0.15874875	UDP	0.77971405	Magnitue	1
Radius	0.06808802	Covariance	0.57125954	Variance	0.85736812
Weight	0.44938718				

### PSO parameter selection

**Figure 5:**
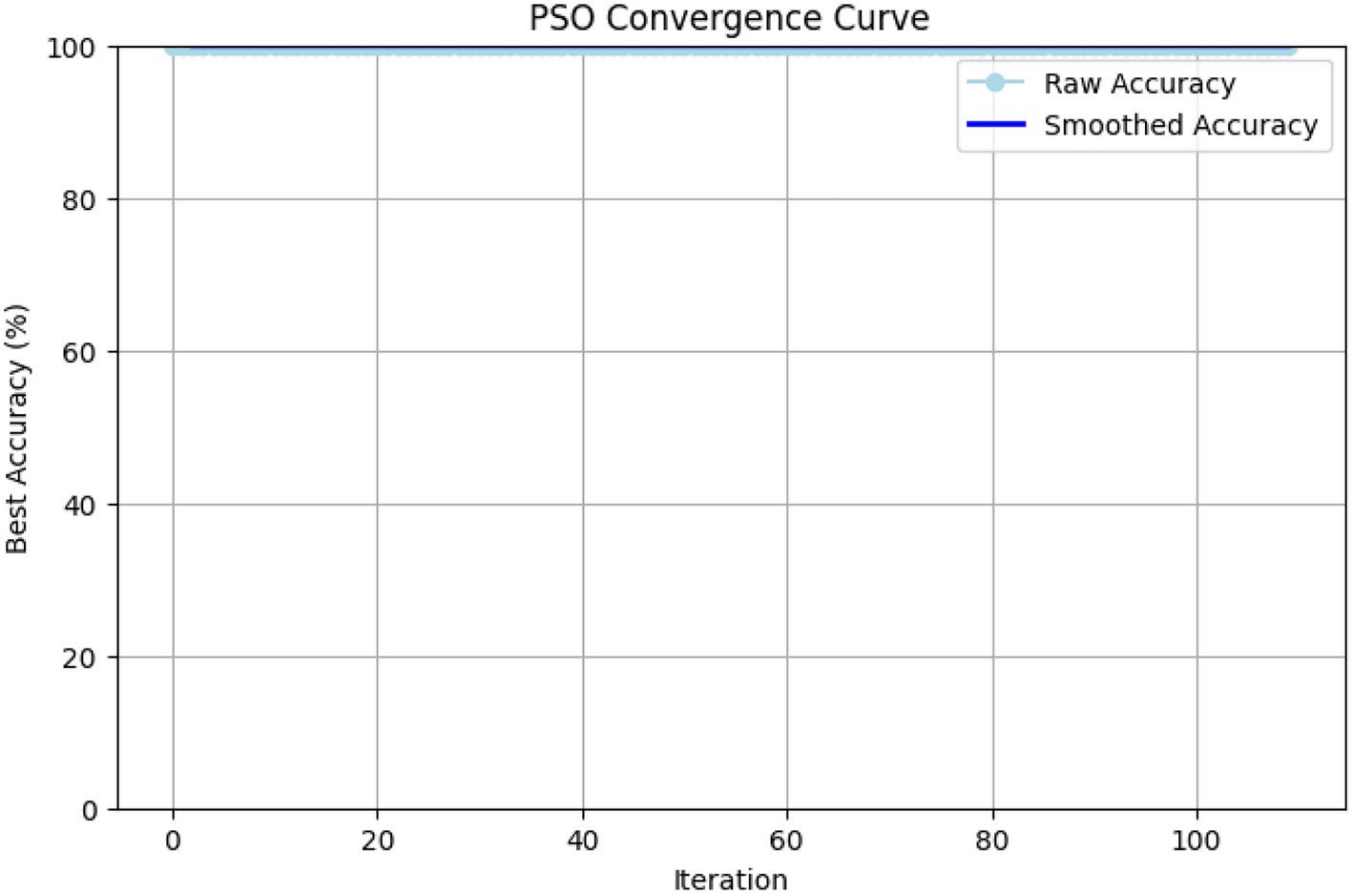
PSO convergence curve for feature selection.

The choice of parameters such as swarm size and the number of iterations directly impacts the balance between the quality of the feature subset found and the computational cost of the search process. In our framework, we selected a swarm size of 100 and a maximum of 10 iterations. This decision was based on established practices and the specific goals of our study. A larger swarm size of 100 was chosen to ensure a comprehensive exploration of the high-dimensional feature space, reducing the risk of the algorithm prematurely converging on a local optimum. The number of iterations was deliberately limited to 10 as a trade-off to maintain computational efficiency. Since our goal is a lightweight and practical framework, an exhaustive, timeconsuming search process was deemed counter-productive. The effectiveness of this choice is demonstrated by the algorithm’s convergence curve, shown in Fig. 5. The convergence curve plots the best fitness score (accuracy) found by the swarm as the search progresses. As illustrated, the algorithm identifies a near-optimal solution with an accuracy of 99.97% almost immediately and shows no further improvement in subsequent iterations. This rapid convergence provides strong evidence that 10 iterations are sufficient to yield a high-quality feature subset without incurring unnecessary computational overhead. This aligns perfectly with our goal of balancing high detection accuracy with efficient deployment.

### Classification models

The standalone classification models DT and KNN were ensembled using three approaches, namely bagging, boosting and Random subspace. In the case of boosting, two variations were concentrated, namely AdaBoost and RUSBoost. 5-fold cross-validation was utilized to validate the proposed framework. The models were trained using the feature subset obtained as a result of feature selection from the proposed PSO-DT algorithm. The results obtained are finally compared and validated.

#### Bagging-based decision tree classifier

Initially, the best solution obtained from the PSO-DT algorithm is taken and masked to be either ‘0’ or ‘1’. When the feature is masked as 1, the feature is considered for training, and when it is masked as 0, it is omitted. A threshold of 0.5 is maintained for the binary mask.

**Table 8:** Classification report: PSO-DT based bagging DT classifier.

Target class	Precision	Recall	F1 score	Support
DDoS-ACK_Fragmentation	0.99	0.99	0.99	441
DDoS-HTTP_Flood	0.94	0.98	0.96	48
DDoS-ICMP_Flood	1.00	1.00	1.00	10,925
DDoS-ICMP_Fragmentation	1.00	1.00	1.00	726
DDoS-PSHACK_Flood	1.00	1.00	1.00	6409
DDoS-RSTFINFlood	1.00	1.00	1.00	6131
DDoS-SYN_Flood	1.00	1.00	1.00	6173
DDoS-SlowLoris	0.83	0.95	0.89	21
DDoS-SynonymousIP_Flood	1.00	1.00	1.00	5436
DDoS-TCP_Flood	1.00	1.00	1.00	6982
DDoS-UDP_Flood	1.00	1.00	1.00	8369
DDoS-UDP_Fragmentation	0.99	0.99	0.99	473

**Figure 6:**
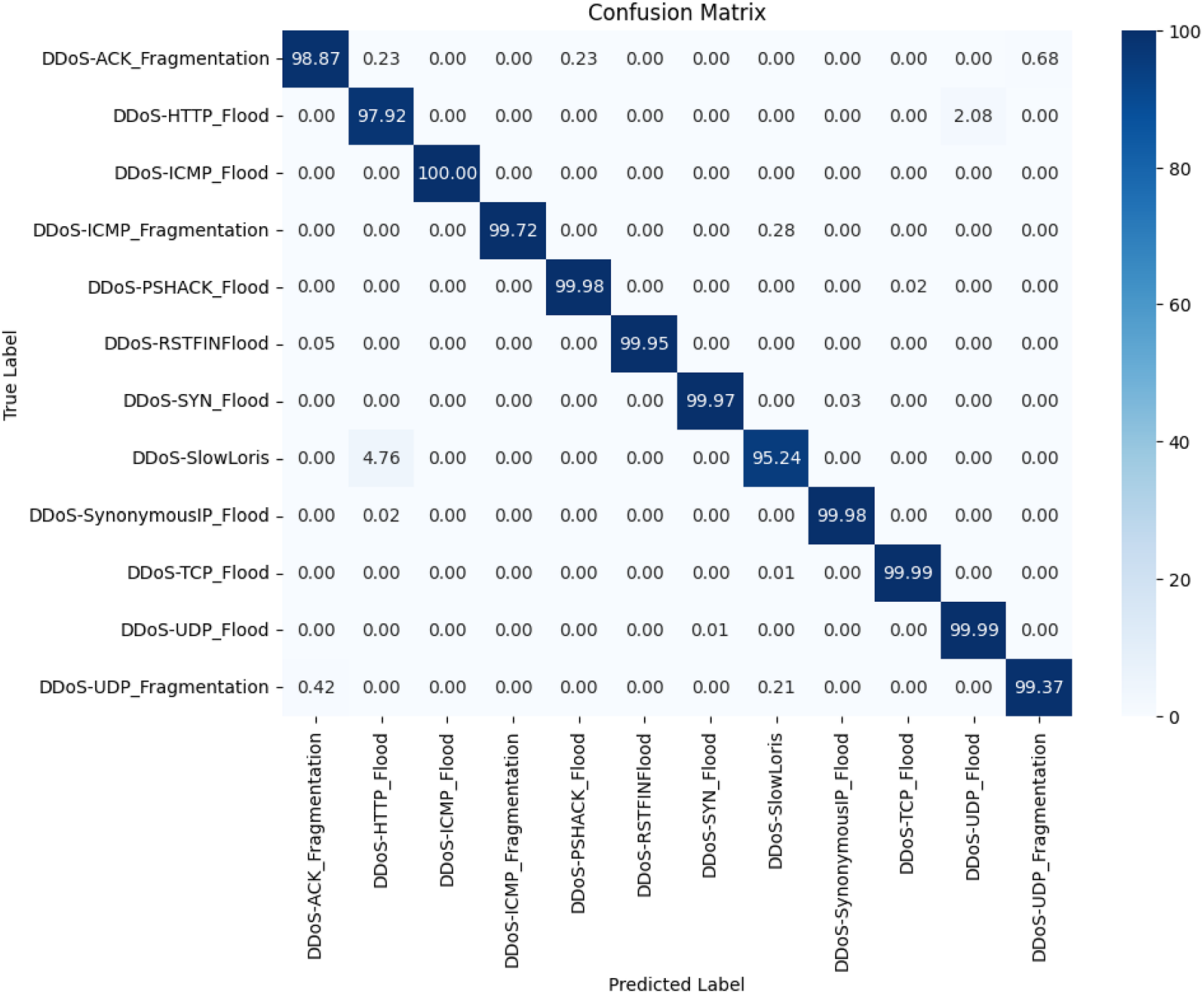
Confusion matrix: proposed PSO-DT based bagging DT classifier.

Then, the final training and testing data sets are reduced by including only the selected features from the binary feature mask. The reduced training set is utilized for training the ensemble of decision trees. Totally 50 decision trees are created and ensembled using the bagging technique. Since faster training is the requirement, all the CPU cores are utilized. The training is then started, and the total time required for training is calculated. Then, using the trained model, the testing data is validated, using which the time required for prediction is calculated in seconds. The classification report is obtained for this model and is illustrated in the Table 8. The confusion matrix for the same is shown in Fig. 6.

The training time for training the parameters and ending with a learned model is 21.22 seconds, the time required for prediction is 0.26 seconds, and the model size is 1.70 MB when the proposed PSO-DT-based Bagging DT Classifier is used. The model achieved an accuracy of 99.96%.

#### AdaBoost-based decision tree classifier

**Figure 7:**
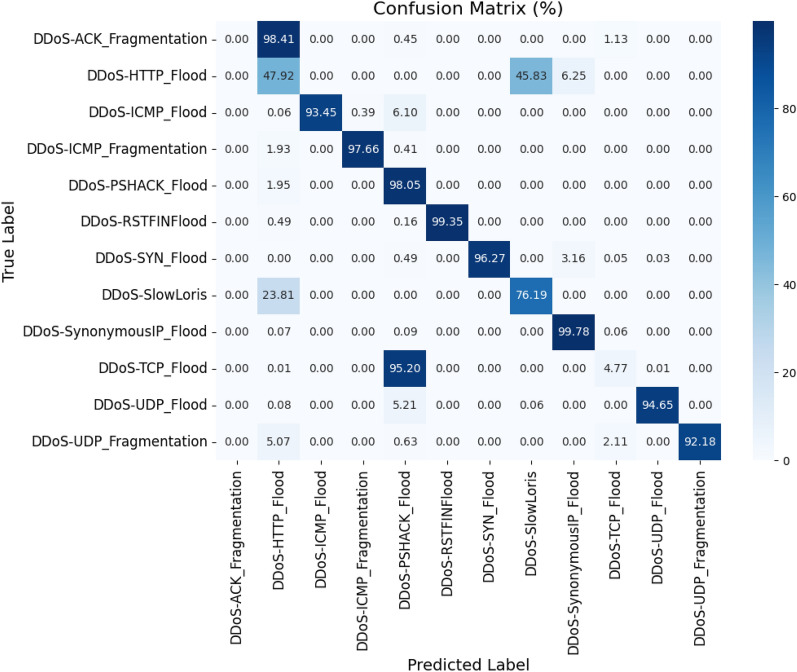
Confusion matrix: proposed PSO-DT based AdaBoost DT classifier.

This model uses several weak learners and combines them to arrive at a final strong classifier. This model functions sequentially and tries to correct the mistakes made in the previous round during the next immediate round. The base model, which is a weak learner, is initially defined. The base model considered here is called a decision stump, which is a decision tree with only one split. The tree is considered to be a shallow tree, and the limit for splitting is fixed to be one level. This tree is too weak when used alone, but the Adaboost ensembling approach would boost the performance of the weak learners. This approach also helps in avoiding overfitting. The model is also initialized with a random state of 42, ensuring that the results produced are almost the same every time. The total number of rounds to boost the weak learners is set at 50, which means that the algorithm will sequentially build 50 weak learners. The learning rate is set to 1.0, which allows more aggressive updates and is faster. Using this model, the training dataset is trained using the selected best features from the proposed PSO-DT feature selection algorithm, and the training time required is calculated. Then the validation data is used for predicting the target classes, and the prediction time is also recorded. The results obtained are shown by the confusion matrix in Fig. 7.

The total training time is 34.4733 seconds, and the prediction time is 0.6416 seconds. The model deployment size is 0.0667 MB. The evaluation metrics for the classification performed using this variation of the PSO-DT based Adaboost DT are tabulated in the Table 9.

**Table 9:** Classification report: PSO-DT based AdaBoost DT classifier.

Target Class	Precision	Recall	F1 Score	Support
DDoS-ACK_Fragmentation	0.00	0.00	0.00	441
DDoS-HTTP_Flood	0.03	0.48	0.06	48
DDoS-ICMP_Flood	1.00	0.93	0.97	10925
DDoS-ICMP_Fragmentation	0.94	0.98	0.96	726
DDoS-PSHACK_Flood	0.45	0.98	0.61	6409
DDoS-RSTFINFlood	1.00	0.99	1.00	6131
DDoS-SYN_Flood	1.00	0.96	0.98	6173
DDoS-SlowLoris	0.37	0.76	0.50	21
DDoS-SynonymousIP_Flood	0.96	1.00	0.98	5436
DDoS-TCP_Flood	0.94	0.05	0.09	6982
DDoS-UDP_Flood	1.00	0.95	0.97	8369
DDoS-UDP_Fragmentation	1.00	0.92	0.96	473

#### RUSBoost based decision tree classifier

**Figure 8:**
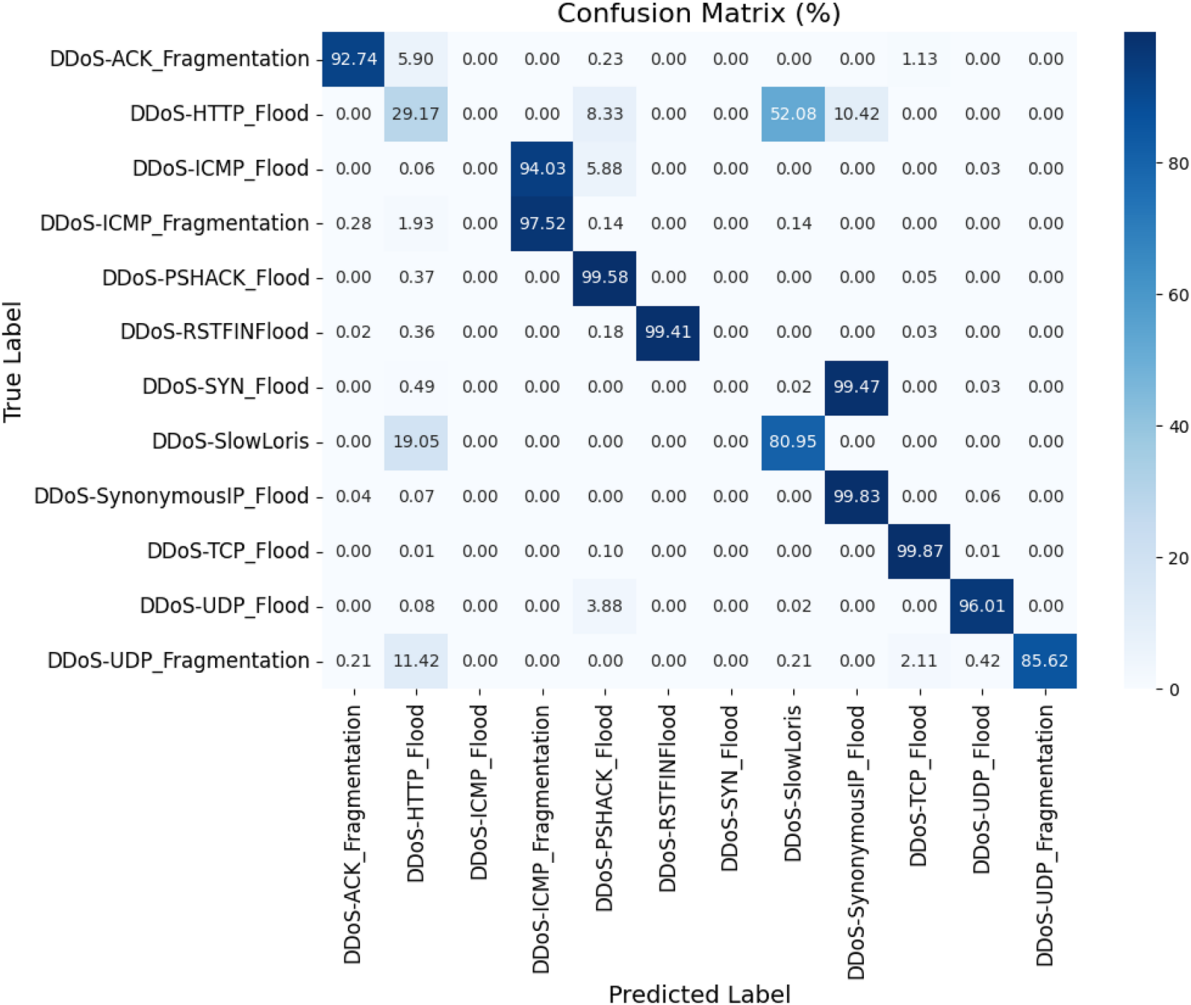
Confusion matrix: proposed PSO-DT based RUSBoost DT classifier.

This variation is built using a combination of random undersampling and Adaboost. In this certain classes are removed randomly for balancing the distribution of the samples. The base weak learner used is the decision tree stump with one split. 50 weak learners are initialized to learn the ensemble model. The learning rate is set to 1.0, and hence it is faster. The random state is initialized as 42, similar to the other variants. Then the model is trained using the subset of the training data retrieved from the whole based on the selected features as a result of PSO-DT. The total time required for training is estimated as 98.6793 seconds. Then, using this, the target classes are predicted for the validation data, and the time required for this is 0.6566 seconds. The size of the deployment model is 117.4072 MB. The confusion matrix obtained as a result of this model is shown in Fig. 8.

The precision, recall, F1 score and support values for the classification results concerning each target class are tabulated in the Table 10. This classification model achieved an accuracy of 66.11%.

**Table 10:** Classification report: PSO-DT based RUSBoost DT classifier.

Target class	Precision	Recall	F1 Score	Support
DDoS-ACK_Fragmentation	0.99	0.93	0.96	441
DDoS-HTTP_Flood	0.07	0.29	0.11	48
DDoS-ICMP_Flood	0.00	0.00	0.00	10925
DDoS-ICMP_Fragmentation	0.06	0.98	0.12	726
DDoS-PSHACK_Flood	0.87	1.00	0.93	6409
DDoS-RSTFINFlood	1.00	0.99	1.00	6131
DDoS-SYN_Flood	0.00	0.00	0.00	6173
DDoS-SlowLoris	0.36	0.81	0.50	21
DDoS-SynonymousIP_Flood	0.47	1.00	0.64	5436
DDoS-TCP_Flood	1.00	1.00	1.00	6982
DDoS-UDP_Flood	1.00	0.96	0.98	8369
DDoS-UDP_Fragmentation	1.00	0.86	0.92	473

#### Random subspace ensemble using KNN bagging classifier

**Figure 9:**
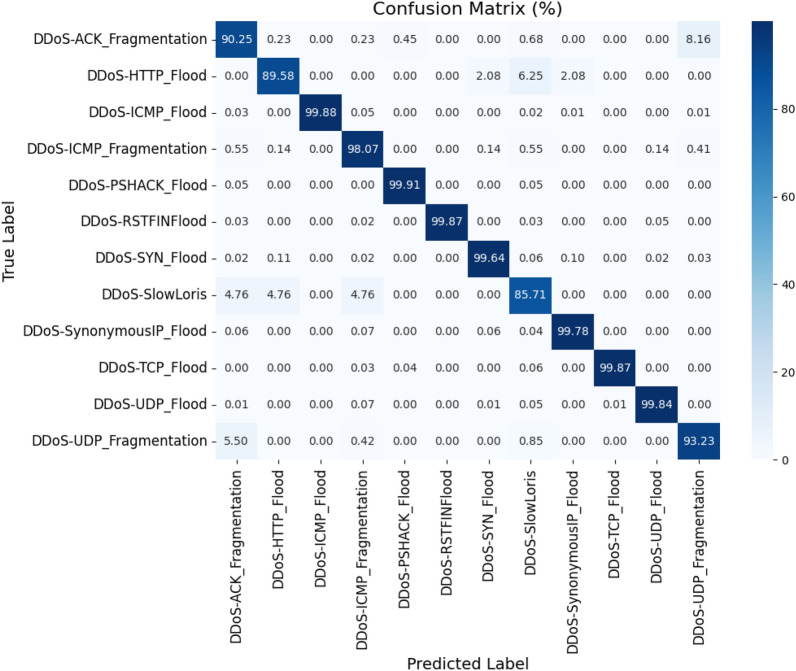
Confusion matrix: proposed PSO-DT based random subspace KNN bagging classifier.

The selected features are used for training and testing the model. The model uses a KNN-based learner, and the final predictions are aggregated using majority voting. The random subspace is initialized to be 0.7, which instructs the model to choose only 70% of the features for the ensemble randomly each time. This helps in introducing diversity among the base models. The training is performed using 50 KNN models, each with a different subset of features, by random sampling. Similarly to all the other variations, the random state is set to 42, and all CPU cores are utilized for training. When the training is performed using this configuration, the total time required for training the data is estimated to be 21.7785 seconds. The inference time required to predict the target classes is 707.5585 seconds. The prediction result obtained can be interpreted from the confusion matrix shown in Fig. 9. The classification results can be evaluated using the metrics precision, recall, F1 score and support value as tabulated in the Table 11. The model achieved an accuracy of 99.65% and the size of the model during deployment would be 3472.5218 MB.

**Table 11:** Classification Report: PSO-DT based Random Subspace KNN Classifier.

Target class	Precision	Recall	F1 Score	Support
DDoS-ACK_Fragmentation	0.90	0.90	0.90	441
DDoS-HTTP_Flood	0.81	0.90	0.85	48
DDoS-ICMP_Flood	1.00	1.00	1.00	10925
DDoS-ICMP_Fragmentation	0.97	0.98	0.97	726
DDoS-PSHACK_Flood	1.00	1.00	1.00	6409
DDoS-RSTFINFlood	1.00	1.00	1.00	6131
DDoS-SYN_Flood	1.00	1.00	1.00	6173
DDoS-SlowLoris	0.34	0.86	0.49	21
DDoS-SynonymousIP_Flood	1.00	1.00	1.00	5436
DDoS-TCP_Flood	1.00	1.00	1.00	6982
DDoS-UDP_Flood	1.00	1.00	1.00	8369
DDoS-UDP_Fragmentation	0.91	0.93	0.92	473

### Statistical significance analysis

To provide a rigorous, quantitative validation of our model choices, we conducted a series of paired t-tests on the 5-fold cross-validation accuracy scores of our key classifiers. This analysis formally assesses whether the observed performance differences are statistically significant or merely due to chance. Our null hypothesis (H) for each test was that there is no significant difference in the mean accuracy between the two models being compared, using a significance level of $\hbox {p } < 0.05$. First, we compared our proposed Bagging DT (BagDT) ensemble against a standalone Decision Tree (DT) to evaluate the contribution of the ensembling technique itself. Subsequently, we compared the BagDT model against the alternative boosting-based ensembles, RUSBoost and AdaBoost, to validate its superiority. The results are summarized in Table 12. The statistical analysis yielded two critical insights. First, the t-test between the standalone DT and the BagDT ensemble showed no statistically significant difference in accuracy (p = 0.2114). This is a powerful testament to the effectiveness of our PSODT feature selection, which identifies a feature subset so discriminative that even a simple model achieves near-perfect results. In this high-performance context, the primary role of the Bagging ensemble is to provide model robustness and stability by reducing variance, a crucial trait for real-world deployment. Second, the comparisons between BagDT and the boosting variants are definitive. With p-values of 0.0020 (vs. RUSBoost) and 0.0024 (vs. AdaBoost), the results show that the Bagging DT model is statistically significantly more accurate than its boosting-based counterparts. This provides conclusive evidence that our chosen approach is the superior one among the tested ensemble methods, validating its selection for the final framework.

**Table 12:** Paired T-test results for model comparisons (accuracy).

Comparison pair	Mean accuracy	t-statistic	p-value	Result ($\hbox {p} < 0.05$)
Standalone DT	99.98% ± 0.01%	1.4863	0.2114	Not Significant
Bagging DT (Ours)	99.98% ± 0.01%			
Bagging DT (Ours)	99.98% ± 0.01%	-7.2123	0.0020	Significant
RUSBoost	79.94% ± 5.56%			
Bagging DT (Ours)	99.98% ± 0.01%	-6.8207	0.0024	Significant
AdaBoost	78.95% ± 6.16%			

### Discussion on results

Based on the experimental analysis performed, it is clear that the BagDT Ensemble Model based on the proposed PSO-DT integrated hybrid framework performs better compared to traditional state-of-the-art models, other variants of the ensemble, and the MRMR-PCA BagDT model^28^ proposed by us in our previous work in terms of precision. The comparative analysis of the proposed framework with the state-of-the-art literature techniques can be interpreted from Table 13 given below. The table clearly shows that the proposed model outperforms all the state-of-the-art related works.

The superior performance of the proposed system can be attributed to the effective integration of its core components. The out-performance relative to the other ensemble variants are from fundamental differences in their mechanisms. Our Bagged DT (BagDT) model excels by using bagging, an approach that reduces variance by training decision trees independently on different data subsets. This makes the final model highly robust to noise and outliers within the training data. In contrast, boostingbased models like AdaBoost and RUSBoost, which sequentially focus on misclassified instances, can sometimes overfit to noisy data, leading to lower overall accuracy as seen in our results. While the Random Subspace KNN achieved high accuracy, its massive model size and extremely high prediction delay render it impractical for realtime IoT deployment. Furthermore, as shown in the comparative analysis in Table 13, the proposed framework outperforms published state-of-the-art models primarily through its holistic focus on both accuracy and deployment readiness. All models in this study were evaluated for their multi-class classification capabilities, but our novel PSO-DT feature selection provides a critical advantage by significantly reducing model complexity and training time without compromising performance, a balance not often prioritized in other works.

The proposed hybrid PSO-DT based BagDT Ensemble model outperforms the other ensemble variants in terms of computational aspects and the deployment model size is lesser than others and is illustrated in Table 14.

To specifically address the model’s real-time capabilities and performance under high traffic loads, we analyzed two key latency metrics: Average Detection Delay and Prediction Speed. As shown in Table 15, our proposed PSO-DT BagDT model achieves an ultra-low detection delay of just 4.7 microseconds per packet. This nearinstantaneous classification is essential for timely threat mitigation. Furthermore, the Prediction Speed metric directly addresses the model’s performance in high-traffic IoT environments. With the ability to process over 212,000 observations per second, our model’s throughput significantly exceeds the data rates of most IoT gateways. This ensures that the detection system will not become a network bottleneck, even during peak traffic or a large-scale attack, validating its suitability for demanding real-world deployment.

**Table 13:** Performance analysis-proposed model vs state-of-the-art techniques.

Ref.	Feature selection	Multi class	Class imbalance handled	Deployment ready	Scalable	Time and speed	Accuracy (%)
^2^	$\times$	$\checkmark$	$\times$	$\times$	$\times$	$\times$	98
^3^	$\times$	$\checkmark$	$\times$	$\times$	$\times$	$\times$	96
^4^	$\times$	$\checkmark$	$\times$	$\times$	$\times$	$\times$	97
^5^	$\checkmark$	$\checkmark$	$\times$	$\times$	$\times$	$\times$	98.96
^6^	$\times$	$\times$	$\times$	$\times$	$\times$	$\times$	99
^7^	$\checkmark$	$\checkmark$	$\times$	$\times$	$\times$	$\checkmark$	Variable
^8^	$\checkmark$	$\checkmark$	$\times$	$\times$	$\times$	$\checkmark$	99.45
Proposed	$\checkmark$	$\checkmark$	$\checkmark$	$\checkmark$	$\checkmark$	$\checkmark$	**99.96**

**Table 14:** Performance of proposed PSO-DT hybrid ensemble models.

	Training time (s)		Prediction speed observations per sec)		
Model	All features	PSO-DT	All features	PSO-DT	Accuracy (%)
AdaBoost	399.17	34.4733	29,000	81,259	83.23
RUSBoost	205.33	98.6793	22,000	79,398	66.11
BagDT	436.47	19.6790	29,000	2,12,432	99.96
Random Subspace KNN	4843.1	21.7785	180	730	99.65

**Table 15:** High-traffic performance of proposed PSO-DT hybrid ensemble models.

Model	Average detection delay (micro seconds)	Model size (MB)	Prediction speed (obs/s)
AdaBoost	12.3	0.07	81,259
RUSBoost	12.6	117.41	79,398
BagDT	**4.7**	**1.70**	**212,432**
Random Subspace KNN	13571.4	3472.52	730

The major contributions of the proposed hybrid framework for detecting DDoS attacks in an IoT environment are as follows.

**Figure 10:**
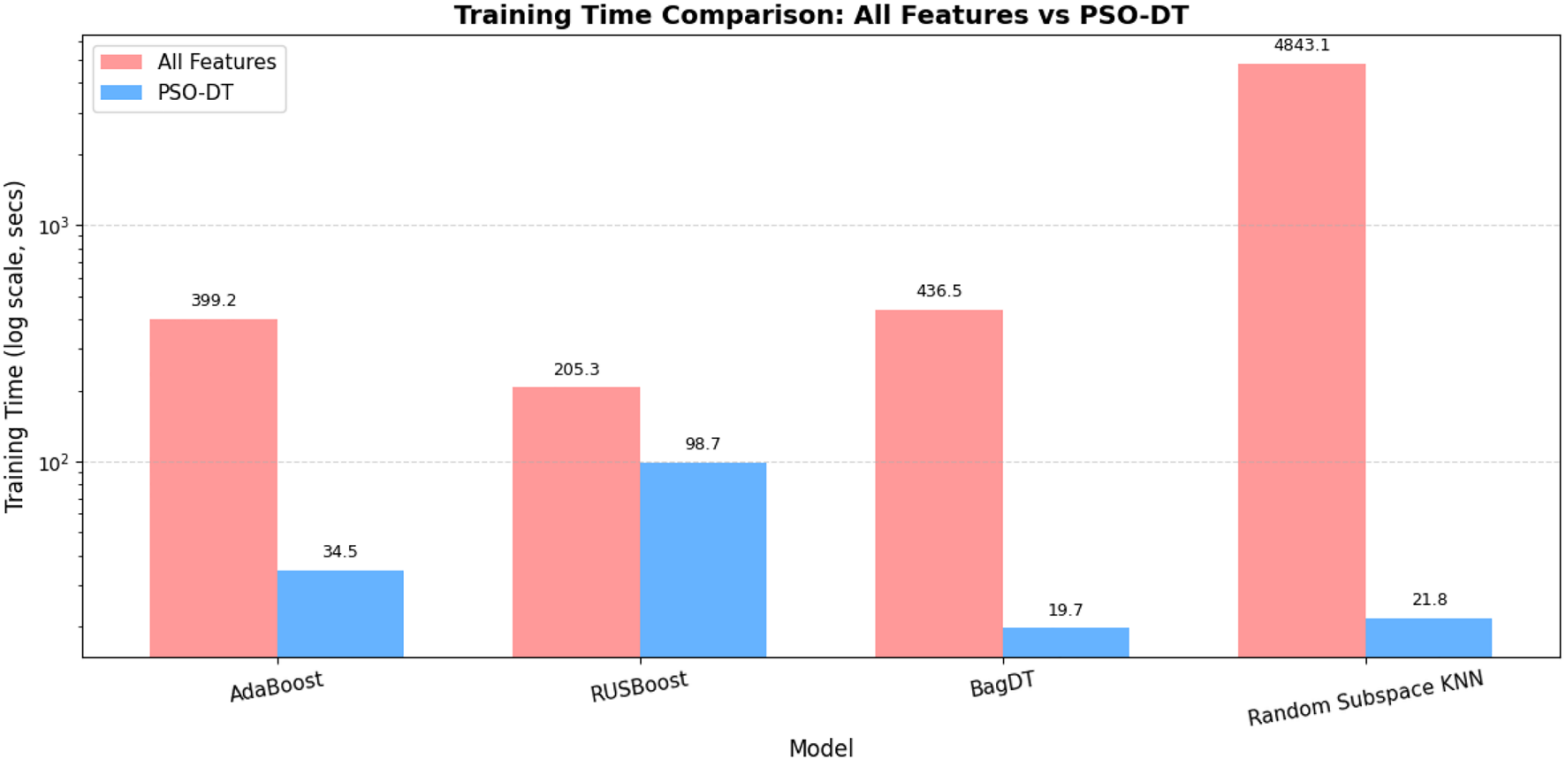
Training time comparison: ALL features vs PSO-DT for ensemble variants.

**Figure 11:**
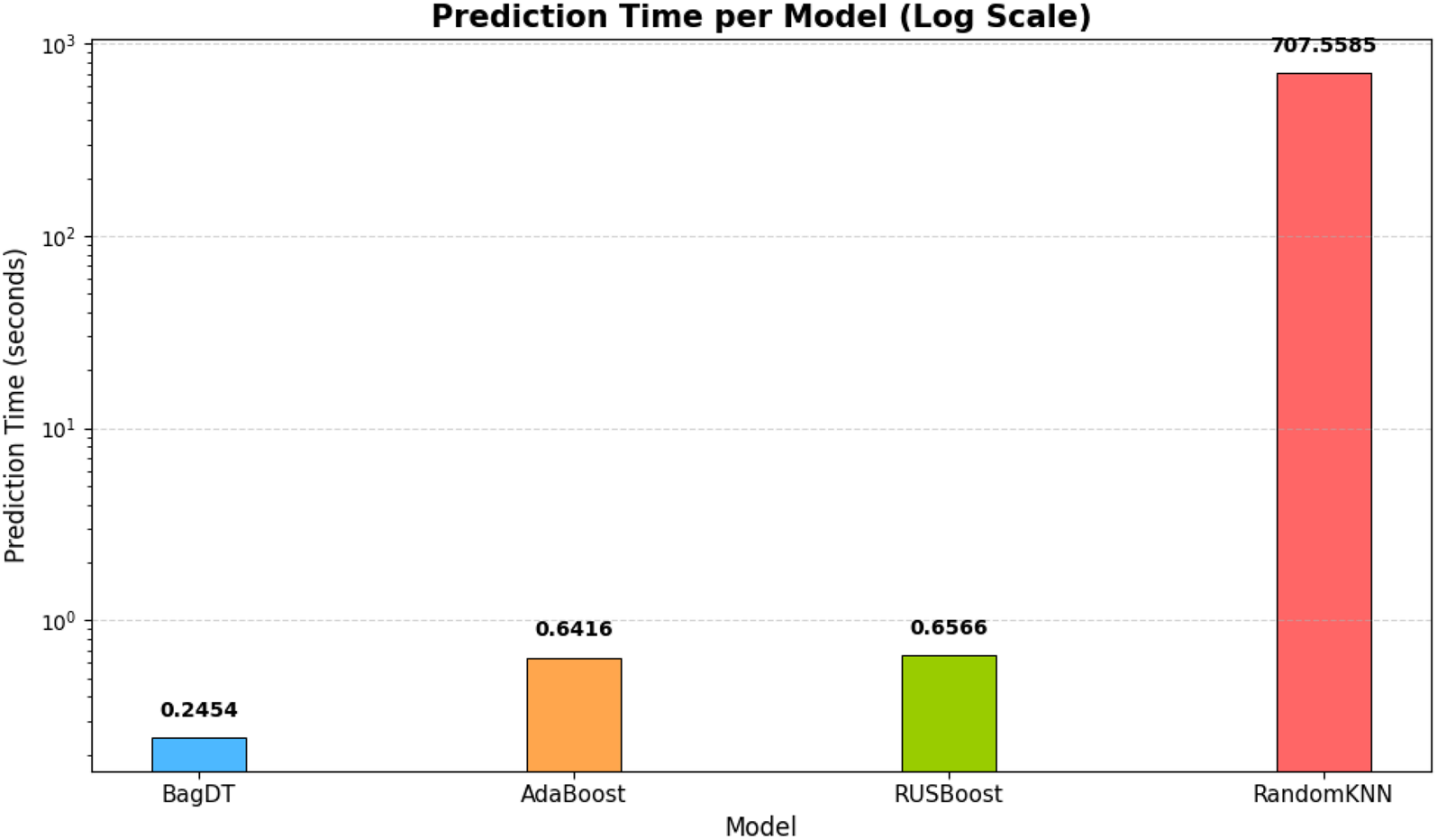
Prediction time analysis: PSO-DT based ensemble variants.

**Figure 12:**
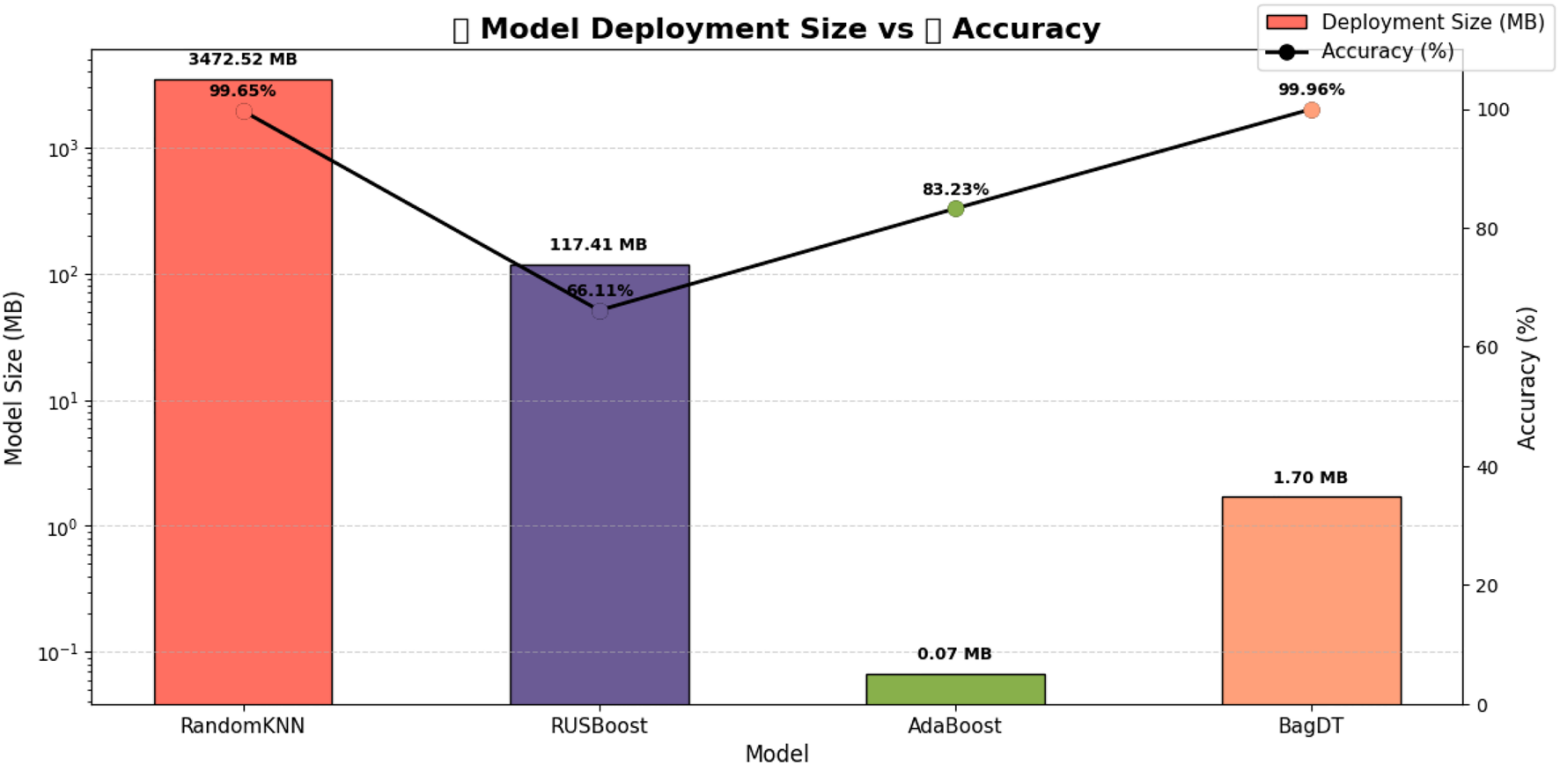
Model deployment size vs accuracy plot: tradeoff.

- The hybrid framework includes an integrated preprocessing phase, which includes all the steps for handling the class imbalance issues as well as automatic encoding and normalization. The SMOTE is an oversampling technique that creates synthetic samples for the minority class by interpolating between existing minority instances. This reduces overfitting compared to random oversampling. This also improves minority class recall. Since the focus was to have a balance between performance and control, this is the best technique to be used in a real-time environment.
- The proposed PSO-DT feature selection algorithm reduces the feature space by avoiding the need for separate dimensionality reduction techniques like Principal Component Analysis. This not only reduces the dimension but also improves the performance of the model. Since PSO is metaheuristic, it can easily adapt to different datasets and classifiers.
- Among the variants of the ensemble models, the PSO-DT based BagDT requires a lesser training time of 19.679 seconds, as illustrated in the Fig. 10.
- The time required for predicting the occurrence of attack is lesser in the case of the proposed hybrid PSO-DT based BagDT Ensemble model when compared with other variants and can be seen clearly in the Fig. 11.
- Since the prediction model developed has to be deployed in an edge device which is usually size restricted, the size of the model plays a major role and from the Fig. 12 it is evident that the proposed PSO-DT based BagDT is lightweight with better accuracy, lesser training and prediction time.

From the experimental analysis conducted, it is clear that the proposed PSO-DT based BagDT Ensemble framework outperforms all the other variants, namely AdaBoost, RUSBoost and Random Subspace KNN. This interpretation is based on the accuracy and model size. In the case of Random Subspace KNN, the accuracy is equivalent to that of BagDT, but it would suffer from deployment issues in a real-time IoT environment since the model size is too high. Also, the time required for training is higher in Random Subspace KNN when compared to BagDT.

### Discussion on model generalization and robustness

While direct experimentation on additional datasets provides the ultimate proof of generalization and is a valuable direction for future work, the current study includes several key methodological steps and validation results that strongly support our model’s robustness.

**Figure 13:**
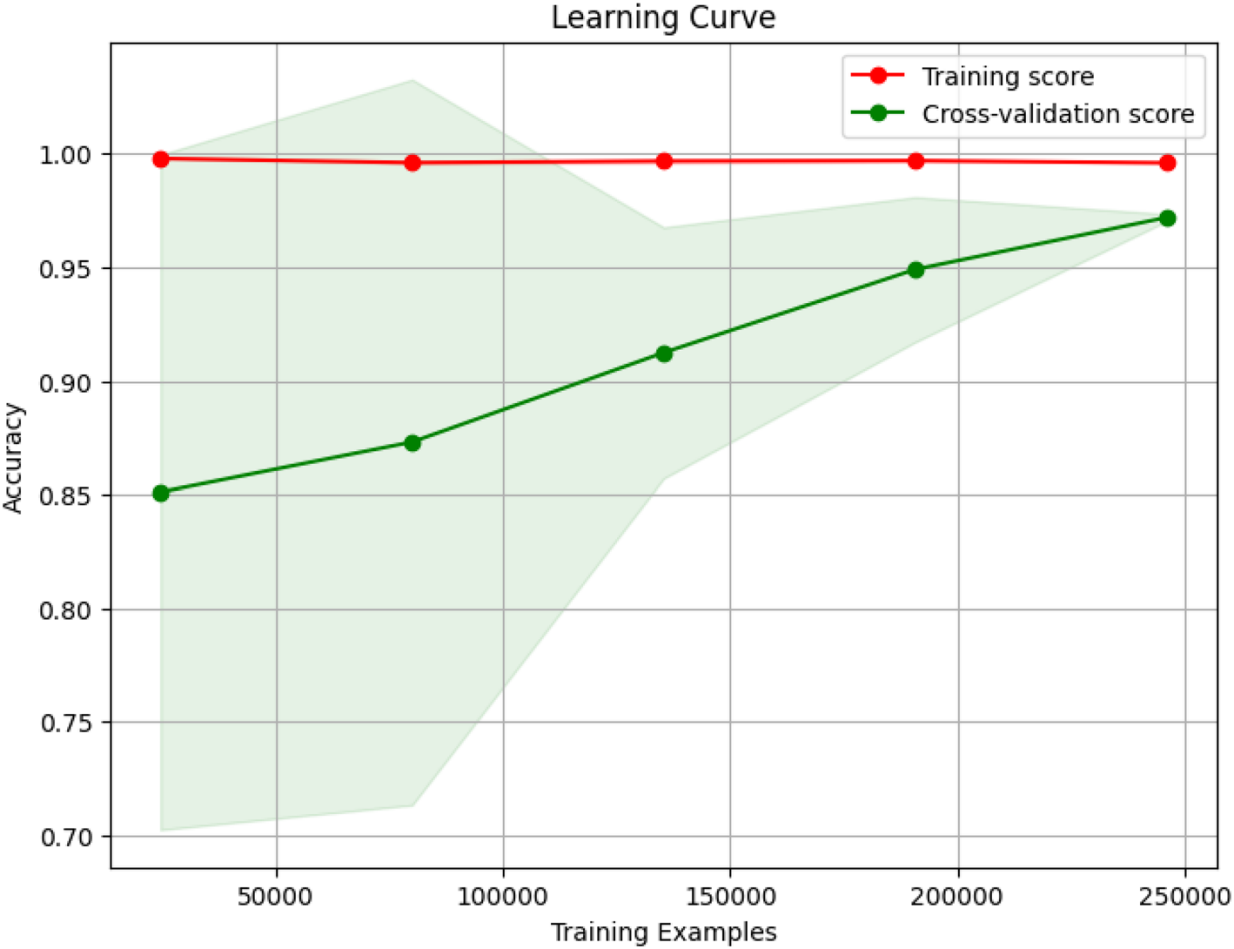
Learning curve for the PSO-DT based BagDT model.

**Figure 14:**
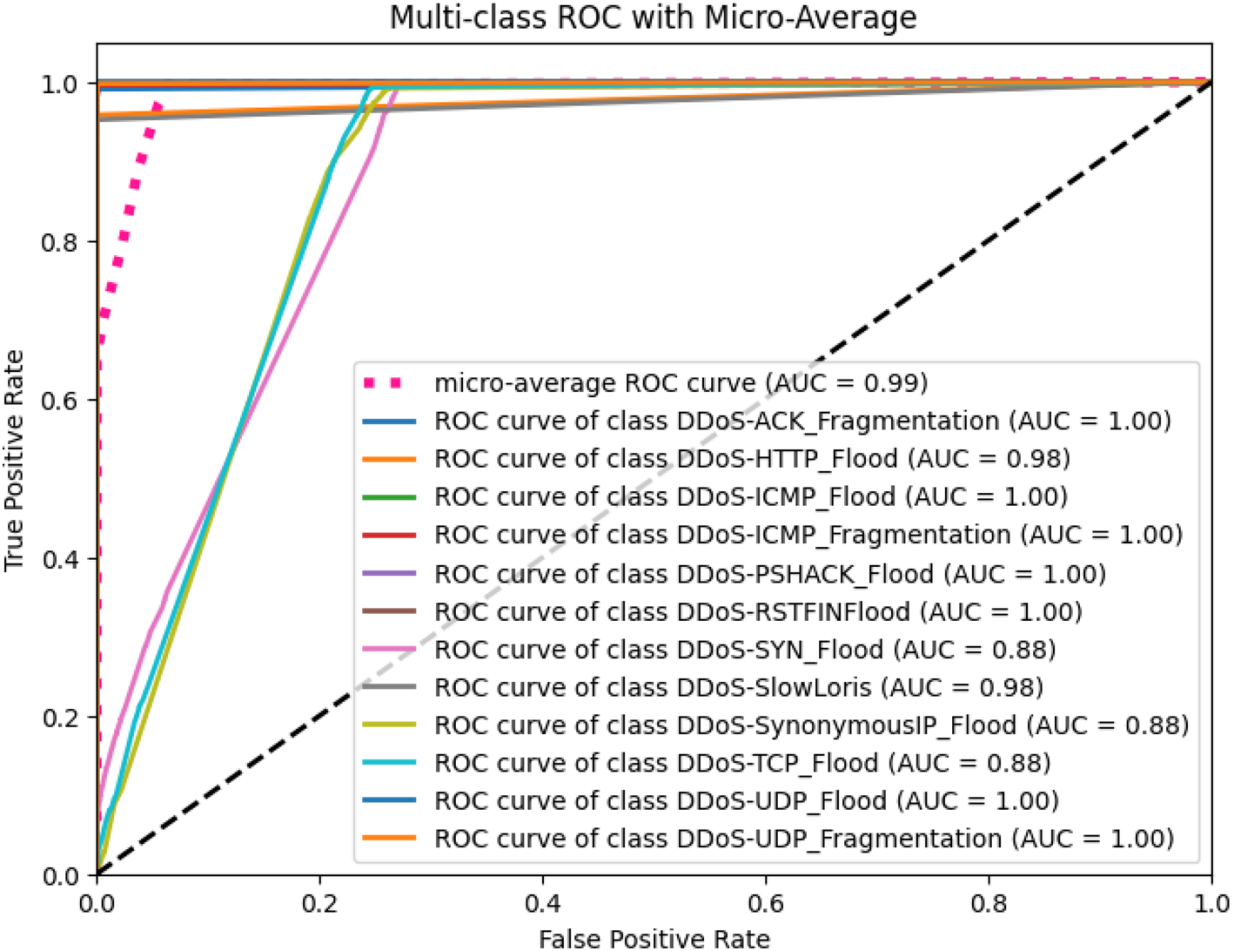
ROC curve for the proposed PSO-DT based BagDT model.

#### Visualizing generalization with learning curves

To ensure that the proposed model does not overfit the training data, we plotted its learning curve . This curve compares the training score against the 5-fold cross-validation score on unseen data as the number of training samples increases. As shown in Fig. 13, the training and cross-validation scores converge to a high level of accuracy. The minimal gap between the two curves indicates that the model generalizes well to new data and does not suffer from high variance or overfitting. This is a critical validation of our model’s stability.

#### Evaluating classifier robustness with ROC/AUC

To further evaluate the model’s robustness, we analyzed the Receiver Operating Characteristic (ROC) curve and the Area Under the Curve (AUC), as shown in Fig. 14. All classification thresholds. Our proposed model achieves a near-perfect micro-average AUC of 0.99, confirming its outstanding ability to discriminate between different attack classes and benign traffic. In addition to these visual analytics, our framework’s robustness is supported by the following list.

$\bullet$ Rigorous Internal Validation: The model’s performance was not evaluated on a simple train-test split alone. We employed 5-fold cross-validation during the feature selection and evaluation phases. This technique repeatedly tests the model on different subsets of the data, providing a more reliable estimate of its performance on unseen data and mitigating the risk of overfitting to a specific test set.

$\bullet$ Fundamental Nature of Selected Features: Our PSO-DT algorithm selected features such as packet rates, flag counts, and protocol types. These are not arbitrary characteristics of a single dataset but are fundamental indicators of network behavior and DDoS attacks (e.g., a flood of SYN packets is a universal signature). A model trained on these core features is inherently more likely to be robust across different network environments.

$\bullet$ Comprehensiveness of the CICIoT2023 Dataset: The primary dataset used in this study, CICIoT2023, is a modern, large-scale dataset generated from a complex IoT environment with 150 devices and a wide variety of DDoS attack types. Its diversity and realism make it a strong benchmark for evaluating model performance in a way that is already indicative of real-world applicability.

In summary, while we fully agree that testing on additional public datasets such as Edge-IIoTset or BoT-IoT is an important next step, the rigorous cross-validation, analysis of learning curves, and the fundamental nature of the features selected provide substantial evidence for the generalization capability of our proposed framework.

### Performance analysis on imbalanced data

**Figure 15:**
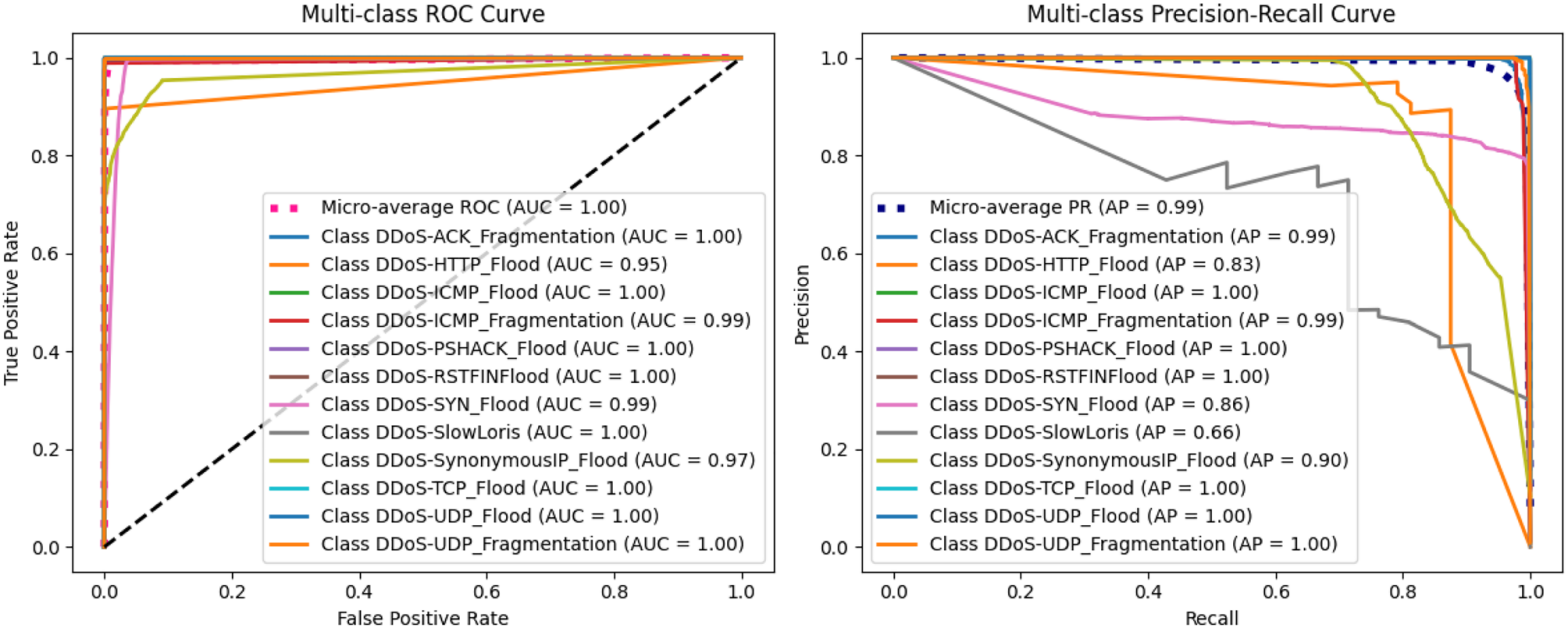
Micro-averaged ROC and precision-recall curves for the proposed model.

While ROC-AUC provides a comprehensive measure of classifier performance, its interpretation can be overly optimistic on the test sets with a severe class imbalance which is a characteristic of our validation data. In such scenarios, a large number of true negatives can inflate the performance score hence masking potential weaknesses in detecting rare positive classes. To provide a more stringent and insightful evaluation, we therefore supplement our analysis with the Precision-Recall (PR) curve. The PR curve is particularly effective here because it evaluates the trade-off between precision (the fraction of positive predictions that are correct) and recall (the fraction of actual positives that are correctly identified), thus focusing directly on the performance of the rare attack categories. Figure 15 presents both the micro-averaged ROC curve and the corresponding PR curve for our proposed model. The Precision-Recall curve on the right of the Fig. 15 provides a stringent performance evaluation. Our model achieves a micro-averaged Area Under the PR Curve (AUPRC) of 0.99, a near-perfect score indicating high real-world effectiveness. This result confirms that the model maintains exceptional precision and recall, minimizing false alarms while detecting most true attacks. This robust performance, validated with a class imbalance-sensitive metric, proves the framework’s reliability for security scenarios where detecting rare threats is critical.

## Conclusion

This research successfully developed a lightweight and efficient prediction model for real-time DDoS attack detection in IoT environments. The novelty of our PSO-DT feature selection algorithm was clearly demonstrated by its ability to intelligently reduce the feature space, leading to a remarkable 95.49 while simultaneously reducing model complexity. This innovative approach eliminates the need for separate dimensionality reduction steps. Consequently, the final PSO-DT based BagDT model achieved an outstanding accuracy of 99.96 this work’s innovative focus on deployment readiness was validated by the final model’s lightweight footprint of just 1.70 MB, making it highly scalable and suitable for deployment on resource-constrained edge devices. By balancing high classification performance with practical operational metrics, our integrated framework provides a robust solution to a critical challenge in IoT security. Future work will involve analyzing the effectiveness of this framework on a wider variety of datasets to further test its adaptability.

## Data Availability

The dataset used in this study, the CICIoT2023 dataset, is publicly available from the Canadian Institute for Cybersecurity (CIC) at the University of New Brunswick. The dataset can be accessed at https://www.unb.ca/cic/datasets/iotdataset-2023.html.
